# Renal cancer: signaling pathways and advances in targeted therapies

**DOI:** 10.1002/mco2.676

**Published:** 2024-08-01

**Authors:** Aimin Jiang, Jinxin Li, Ziwei He, Ying Liu, Kun Qiao, Yu Fang, Le Qu, Peng Luo, Anqi Lin, Linhui Wang

**Affiliations:** ^1^ Department of Urology Changhai Hospital Naval Medical University Shanghai China; ^2^ Department of Urology Jinling Hospital Affiliated Hospital of Medical School Nanjing University Nanjing China; ^3^ Department of Oncology Zhujiang Hospital Southern Medical University Guangzhou Guangdong China

**Keywords:** molecular mechanisms, precision medicine, renal cancer, signaling pathways, targeted therapy

## Abstract

Renal cancer is a highlyheterogeneous malignancy characterized by rising global incidence and mortalityrates. The complex interplay and dysregulation of multiple signaling pathways,including von Hippel–Lindau (*VHL*)/hypoxia‐inducible factor (*HIF*), phosphoinositide 3‐kinase (*PI3K*)/protein kinase B (*AKT*)/mammalian target of rapamycin (*mTOR*), Hippo–yes‐associated protein (*YAP*), Wnt/ß‐catenin, cyclic adenosine monophosphate (*cAMP*), and hepatocyte growth factor (*HGF*)/*c‐Met*, contribute to theinitiation and progression of renal cancer. Although surgical resection is thestandard treatment for localized renal cancer, recurrence and metastasiscontinue to pose significant challenges. Advanced renal cancer is associatedwith a poor prognosis, and current therapies, such as targeted agents andimmunotherapies, have limitations. This review presents a comprehensiveoverview of the molecular mechanisms underlying aberrant signaling pathways inrenal cancer, emphasizing their intricate crosstalk and synergisticinteractions. We discuss recent advancements in targeted therapies, includingtyrosine kinase inhibitors, and immunotherapies, such as checkpoint inhibitors.Moreover, we underscore the importance of multiomics approaches and networkanalysis in elucidating the complex regulatory networks governing renal cancerpathogenesis. By integrating cutting‐edge research and clinical insights, this review contributesto the development of innovative diagnostic and therapeutic strategies, whichhave the potential to improve risk stratification, precision medicine, andultimately, patient outcomes in renal cancer.

## Graphical abstract

Comprehensive summary of therapeutic strategies and corresponding targets in renal cancer.

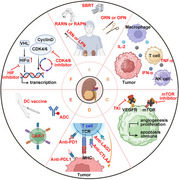

## 1 INTRODUCTION

Renal cancer is a common malignant tumor of the urinary system, with increasing incidence and mortality rates globally. In 2020, there were about 431,000 new cases of renal cancer and 179,000 deaths globally.^1^ The National Cancer Institute predicts 81,000 new cases of kidney and renal pelvis cancer in 2023, representing 4.2% of all new cancer diagnoses. The global incidence of renal cancer continues to escalate, and it is one of the cancers associated with a higher risk of mortality.^2^ Renal cell carcinoma (RCC) is the predominant histological type of renal cancer, accounting for approximately 90% of renal cancer cases. RCC is a heterogeneous urogenital system malignancy and one of the 10 most prevalent neoplasms worldwide, exhibiting the highest mortality rate among urogenital system cancers.^3^, ^4^, ^5^ Recently, the incidence of RCC has risen, with a global recurrence and mortality rate over 40%. RCC is the sixth most common cancer in men and the tenth in women globally.^6^, ^7^, ^8^ RCC is primarily categorized into three subtypes: clear cell RCC (ccRCC), papillary RCC (pRCC), and chromophobe RCC (chRCC). ccRCC is the most prevalent RCC subtype, constituting approximately 70−80% of RCC cases, while pRCC and chRCC account for about 10−15% and 5−10% of cases, respectively.^9^ Additionally, less common RCC subtypes include collecting duct RCC (cdRCC), sarcomatoid RCC, and unclassified RCC (uRCC).^9^ The various RCC subtypes display distinct genetic characteristics, molecular alterations, and prognoses, with ccRCC exhibiting the most aggressive behavior, especially those with venous tumor thrombus.^10^, ^11^, ^12^, ^13^

RCC pathogenesis is complex, involving abnormal activation of multiple signaling pathways and dysregulation of various molecular events. Recently, research on the molecular mechanisms underlying the initiation and progression of RCC has primarily focused on signaling pathways such as von Hippel–Lindau (*VHL*)/hypoxia‐inducible factor (*HIF*), phosphoinositide 3‐kinase (*PI3K*)/protein kinase B (*AKT*)/mammalian target of rapamycin (*mTOR*), p53, cyclic adenosine monophosphate (*cAMP*), and transforming growth factor (TGF)‐β.^2^ Studies have demonstrated that the pivotal molecular alteration in ccRCC is the mutation of the VHL gene, resulting in the constitutive expression of *HIF*, which subsequently activates multiple growth factor pathways, such as vascular endothelial growth factor (*VEGF*) and platelet‐derived growth factor (*PDGF*).^2^ Moreover, genomic instability is also a hallmark of RCC. Genes such as *SETD2*, *PBRM1*, *BAP1*, *MTOR*, and *KDM5C* are frequently mutated in RCC.^2^ In addition to *VHL* pathway abnormalities, non‐ccRCC subtypes may also harbor mutations in other genes such as *CDKN2A*, *PTEN*, *NRF2*, *TP53*, *TFE3*, *TFEB*, and *SMARCB1*.^2^ Renal cancer exhibits several distinctive features in its signaling pathways compared with other malignant neoplasms. For example, the *VHL*–*HIF* axis plays a pivotal role in renal cancer pathogenesis, with *VHL* inactivation and constitutive HIF activation driving angiogenesis and metabolic reprogramming. This distinctive feature differentiates renal cancer from many other solid malignancies and has led to the development of *HIF*‐targeted therapies. Moreover, the *PI3K*/*AKT*/*mTOR* pathway is frequently hyperactivated in renal cancer, often in conjunction with VHL–HIF signaling, creating a distinctive therapeutic vulnerability. Similarly, the Hippo–*YAP* pathway also displays distinctive alterations in renal cancer, with *YAP*/*TAZ* activation contributing to tumor growth and metastatic dissemination. Exploiting these pathway‐specific aberrations has the potential to enhance the precision and efficacy of targeted therapies for renal cancer. Therefore, elucidating the regulatory mechanisms of renal cancer‐related signaling pathways in depth is crucial for identifying new therapeutic targets and developing novel treatment strategies.

RCC is one of the most invasive and lethal malignant neoplasms of the urinary system. Although surgical resection is the primary treatment modality for localized RCC, a substantial proportion of patients still experience recurrence and metastasis following surgery.^9^, ^14^, ^15^, ^16^, ^17^ The prognosis of advanced RCC is poor, primarily attributed to its low sensitivity to radiotherapy and chemotherapy, and its tendency to develop treatment resistance.^9^, ^18^, ^19^ Recently, molecular targeted agents, particularly tyrosine kinase inhibitors (TKIs), have significantly improved the prognosis of patients with advanced RCC^20^; however, common issues such as drug resistance and adverse reactions have become increasingly prominent. Immunotherapies, such as immune checkpoint inhibitors (ICIs), have demonstrated favorable efficacy in the treatment of RCC; however, only a subset of patients can derive benefit from these agents.^9^ In recent years, the combination of ICIs and TKIs as a first‐line treatment regimen has brought new hope for the treatment of advanced RCC.^20^, ^21^ Therefore, there is an urgent need to discover novel therapeutic targets and develop innovative treatment strategies based on the research of RCC pathogenesis to further enhance the therapeutic efficacy and improve patient prognosis. Elucidating the regulatory mechanisms of RCC‐related signaling pathways not only helps to unravel the molecular basis of RCC occurrence and progression but also provides critical insights for the diagnosis, prognostic assessment, and targeted therapy of RCC.

Despite recent advancements, numerous research directions in the field of targeted therapy for renal cancer signaling pathways remain to be explored. In this review, we elucidate the molecular mechanisms underlying signaling pathway aberrations in the pathogenesis and progression of renal cancer, with a particular emphasis on the crosstalk and synergistic interactions between distinct signaling pathways. Moreover, we provide an overview of current and emerging therapeutic strategies and modalities in the management of renal cancer, with the goal of offering additional treatment options for patients with this malignancy. Furthermore, integrating targeted therapy directed at signaling pathways with other therapeutic modalities represents a promising strategy for enhancing outcomes in patients with renal cancer. In conclusion, continued investigation into the regulatory mechanisms governing renal cancer‐associated signaling pathways and the development of novel therapeutic strategies have the potential to confer substantial benefits to patients with renal cancer and yield new insights into the biology of this malignancy.

## 2 SIGNALING PATHWAYS IN RENAL CANCER

**FIGURE 1:**
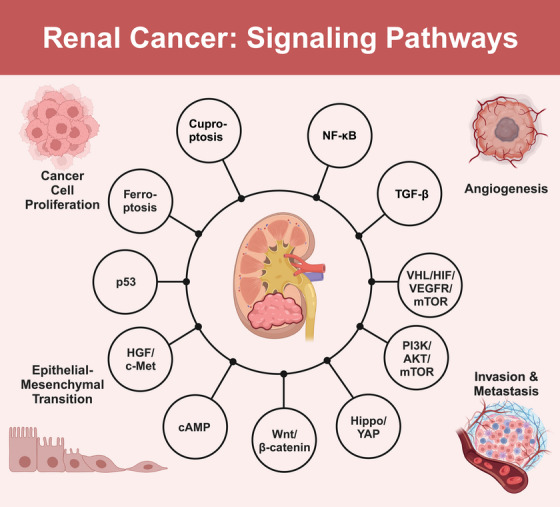
Schematic representation of the critical signaling pathways implicated in the pathogenesis and targeted therapy of RCC. The intricate crosstalk among diverse signaling cascades, including *VHL/HIF*, *PI3K/AKT/mTOR*, Hippo–*YAP*, *Wnt/β*‐catenin, *cAMP*, *HGF*/*c‐Met*, *p53*, ferroptosis‐related, cuproptosis‐related, *NF‐κB*, and *TGF‐β* pathways, plays a pivotal role in the malignant progression of RCC. Elucidating the molecular mechanisms governing these pathways is paramount for devising novel therapeutic strategies and enhancing patient prognosis. This figure was created based on the tools provided by Biorender.com.

The occurrence and progression of renal cancer involve the dysregulation and complex interplay of multiple signaling pathways. Key pathways include *VHL–HIF–VEGFR–mTOR, PI3K/AKT/mTOR*, and *Hippo–YAP*. The *VHL*–*HIF*–*VEGFR*–*mTOR* pathway, driven by *VHL* gene inactivation and subsequent *HIF* accumulation, promotes tumor angiogenesis, cell proliferation, and metastasis in renal cancer. The *PI3K/AKT/mTOR* pathway, frequently hyperactivated in renal cancer, contributes to cancer cell survival, growth, and invasiveness. Notably, there is significant crosstalk between the *VHL–HIF–VEGFR–mTOR* and *PI3K/AKT/mTOR* pathways, forming a complex signaling network that synergistically promotes renal cancer progression. Dysregulation of the Hippo–*YAP* pathway leads to the aberrant activation of *YAP/TAZ* transcriptional coactivators, promoting cell proliferation and inhibiting apoptosis in renal cancer. Additionally, other signaling pathways, such as the *Wnt*/β‐catenin, cAMP, and hepatocyte growth factor (*HGF*)*/c‐Met* pathways, also contribute to the malignant progression of renal cancer. These pathways exhibit intricate interactions and synergistic effects, creating a complex regulatory network that drives renal cancer pathogenesis and progression. In the following subsections, we will delve into the detailed molecular mechanisms and clinical implications of each signaling pathway, providing a comprehensive overview of their roles in renal cancer pathogenesis and progression (Figure 1).

### 2.1 VHL–HIF–VEGFR–mTOR signaling pathway

**FIGURE 2:**
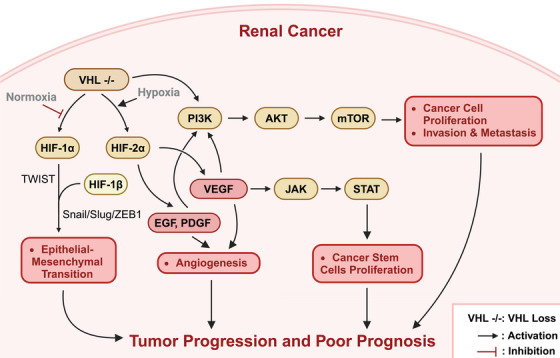
The *VHL–HIF–VEGFR–mTOR* signaling pathway in the pathogenesis of renal cancer. Under normoxic conditions, the von Hippel–Lindau (*VHL*) protein (*pVHL*) recognizes and ubiquitinates hypoxia‐inducible factor‐α (*HIF‐α*), targeting it for degradation by the 26S proteasome. In renal cancer, inactivating mutations or deletions of the VHL gene lead to the accumulation of HIF‐α, which subsequently dimerizes with HIF‐β and translocates to the nucleus. The HIF heterodimer binds to hypoxia response elements (HREs) in the promoter regions of target genes, including vascular endothelial growth factor (*VEGF*), platelet‐derived growth factor (*PDGF*), and carbonic anhydrase IX (*CAIX*), thereby activating their transcription and promoting tumor angiogenesis, proliferation, and metastasis. *VEGF* stimulates the proliferation of vascular endothelial cells and mediates cancer stem cell self‐renewal via the *VEGF* receptor 2 (*VEGFR*‐2)/Janus kinase 2 (*JAK2*)/signal transducer and activator of transcription 3 (*STAT3*) signaling axis. Furthermore, HIF‐1α activates the expression of Twist‐related protein 1 (*TWIST1*) and regulates the Snail/Slug/zinc finger E‐box‐binding homeobox 1 (*ZEB1*) axis, inducing epithelial–mesenchymal transition (EMT) and enhancing the invasive and metastatic potential of tumor cells. The secretion of inflammatory factors, such as transforming growth factor‐β (*TGF‐β*), further exacerbates the EMT process and tumor progression. Moreover, VHL inactivation leads to the hyperactivation of mammalian target of rapamycin (*mTOR*), which is associated with tumor progression and worse prognosis in renal cancer. This figure was created based on the tools provided by Biorender.com.

The *VHL–HIF–VEGFR–mTOR* signaling pathway plays a crucial role in the pathogenesis of renal cancer (Figure 2). The *VHL* gene is one of the most important tumor suppressor genes in renal cancer.^4^ Under physiological conditions, the *VHL* protein (p*VHL*) inhibits angiogenesis and tumor growth, and regulates the stability of *HIF*s.^22^, ^23^ HIF is a heterodimeric transcription factor composed of α and β subunits. The α subunit is stable under hypoxic conditions, while under normoxic conditions, it is recognized and ubiquitinated by the *VHL* E3 ubiquitin ligase complex and ultimately degraded by the 26S proteasome.^4^, ^24^

In ccRCC, the *VHL* gene frequently undergoes inactivating mutations or deletions, resulting in the dysfunction of the *pVHL* and an inability to properly degrade *HIF‐α*, leading to its abnormal accumulation.^25^, ^26^ The accumulated *HIF‐α* combines with *HIF‐β* to form a heterodimer, which enters the nucleus and binds to the promoter regions of genes containing hypoxia response elements, thus activating the transcription of downstream genes, including *VEGF*, *PDGF*, carbonic anhydrase IX (*CAIX*), and others, thereby promoting tumor angiogenesis, proliferation, and metastasis.^22^, ^23^ Among these, *VEGF* not only stimulates the proliferation of vascular endothelial cells but also mediates the self‐renewal of cancer stem cells through the activation of the *VEGFR*‐2/Janus kinase (*JAK*)2/signal transducer and activator of transcription 3 (*STAT3*) signaling axis.^27^ Furthermore, *VHL* inactivation also leads to the overactivation of the *mTOR*, which is associated with tumor progression and a poorer prognosis in ccRCC.^4^ Moreover, in addition to regulating angiogenesis and metabolic reprogramming, the *VHL–HIF* pathway is also closely related to epithelial–mesenchymal transition (EMT).^16^ *HIF‐1α* can activate the expression of Twist‐related protein 1 (*TWIST*1) and regulate the Snail/Slug/*ZEB1* axis, inducing the downregulation of E‐cadherin and promoting the occurrence of EMT, ultimately enabling the tumor cells to acquire a mesenchymal phenotype and enhance their invasive and metastatic capabilities.^28^, ^29^ Simultaneously, this signaling pathway can also stimulate the secretion of inflammatory factors, such as *TGF‐β*, further exacerbating the EMT process and tumor progression.^30^

In summary, the *VHL–HIF–VEGFR–mTOR* signaling pathway plays a crucial regulatory role in the initiation and progression of renal cancer. Inactivation of *VHL* leads to aberrant activation of *HIF*, which in turn causes overexpression of downstream target genes such as *VEGF*, thereby promoting tumor angiogenesis and tumor cell proliferation, and accelerating tumor progression through multiple mechanisms such as EMT and inflammation. Meanwhile, the hyperactivation of the *mTOR* signaling pathway further exacerbates this malignant signaling cascade. Therefore, developing novel antirenal cancer therapeutic strategies targeting this pathway may be highly significant for improving the prognosis of renal cancer patients. Several small molecule inhibitors targeting *HIF*, *VEGFR*, and *mTOR* have entered clinical trial phases, potentially providing new hope for advanced renal cancer patients. Future studies should further elucidate the precise regulatory mechanisms of this pathway, identify new therapeutic targets, and ultimately achieve precise treatment of renal cancer.

### 2.2 PI3K/AKT/mTOR signaling pathway

The *PI3K/AKT/mTOR* signaling pathway is critically involved in the pathogenesis and progression of renal cancer. Abnormal activation of the *PI3K/AKT/mTOR* pathway correlates significantly with the occurrence, invasion, and metastasis of renal cancer. In‐depth research on the regulatory mechanisms of the *PI3K/AKT/mTOR* pathway in renal cancer holds immense potential in elucidating the molecular pathological mechanisms of renal cancer and developing new therapeutic strategies and drug targets. The *PI3K/AKT/mTOR* pathway is an evolutionarily highly conserved signaling pathway that is critically involved in various physiological processes such as cell proliferation, differentiation, apoptosis, and metabolism. *PI3K* is activated by various growth factors and cytokines, which in turn catalyzes the phosphorylation of *PIP2* to generate *PIP3*; PIP3 subsequently recruits *AKT* to the cell membrane and activates it. Activated *AKT* further phosphorylates downstream substrates such as *mTOR*, consequently modulating cell growth, proliferation, and survival.^31^

In RCC, abnormal activation of the *PI3K*/*AKT*/*mTOR* signaling pathway is highly prevalent,^31^ with extracellular signaling molecules and transmembrane receptors playing a pivotal role in initiating the activation of this pathway. In ccRCC, the rate of genetic alterations in *PI3K*/*AKT* pathway components is 27.7%, primarily encompassing changes such as gene amplification, mutation, and deletion.^31^ Inactivation of the *VHL* gene and activation of the HIF signaling pathway are key driving factors in the onset and progression of ccRCC, both of which are intimately interconnected with the *PI3K*/*AKT* signaling pathway, collectively forming an intricate signaling network that facilitates the occurrence and development of ccRCC. In pRCC, the rate of genetic alterations in *PI3K*/*AKT* signaling pathway‐related components is 28%.^31^ Type 1 pRCC is associated with *MET* gene mutations, and activation of *MET* can further lead to the activation of the *PI3K*/*AKT* signaling pathway.^32^ Conversely, type 2 pRCC is associated with fumarate hydratase (*FH*) gene mutations.^33^ chRCC is a rare subtype of RCC; however, its genetic alterations in the *PI3K*/*AKT* signaling pathway are highly significant, with approximately 32% of patients exhibiting mutations or deletions of pathway‐related components.^31^ Recent evidence suggests that the *VHL*/*HIF* and *PI3K*/*AKT* signaling pathways extensively interact within a complex signaling network, thus jointly promoting the occurrence and development of ccRCC. For example, the upregulation of *HIF* expression caused by *VHL* gene inactivation can promote the expression of multiple growth factors, including epidermal growth factor (*EGF*), *PDGF*, and *VEGF*, potentially further activating the *PI3K*/*AKT* signaling pathway. Simultaneously, activation of mammalian target of rapamycin complex 1 (*mTORC1*) and 2 (*mTORC2*) also promotes *HIF* expression, thereby forming a positive feedback loop and leading to sustained activation of the entire signaling network.^31^ Furthermore, studies have also demonstrated that *VHL* gene mutations can promote the progression of ccRCC through a *PI3K/AKT* signaling pathway‐dependent mechanism involving cholesterol ester accumulation.^34^

In RCC, the activation of the *PI3K/AKT/mTOR* signaling pathway can be triggered not only by extracellular signaling molecules and transmembrane receptors but also by alternative mechanisms.^31^, ^35^ For example, glucose deprivation can induce a distinct form of *AKT* phosphorylation and activation in various RCC cell lines. Furthermore, microRNAs (miRNAs), as emerging critical regulators of the *PI3K/AKT* pathway, are garnering significant attention. Numerous studies have demonstrated that miRNAs, including *miR‐148a*, *miR‐182‐5p*, and *miR‐137*, can inhibit the *PI3K/AKT/mTOR* pathway, consequently suppressing the proliferation, migration, and invasive capabilities of RCC cells.^36^ *miR‐148a* targets *AKT2*, inhibiting its expression and activity, subsequently suppressing the activation of downstream *mTOR*, ultimately leading to the inhibition of tumor cell growth. miR‐182‐5p indirectly inhibits *AKT2* activity by targeting *FLOT1*, resulting in increased activity of the downstream transcription factor *FOXO3a*, thereby suppressing tumor cell proliferation.^37^ *miR‐137* directly inhibits the *PI3K/AKT* pathway, leading to increased tumor cell apoptosis, thereby suppressing their growth and metastatic abilities.^38^ In contrast, certain miRNAs, such as *miR‐122*, have the effect of activating the *PI3K/AKT/mTOR* pathway. *miR‐122* indirectly promotes the activation of the *PI3K/AKT/mTOR* pathway by suppressing the expression of tumor suppressor genes, such as *SPRY2*, thereby relieving the inhibition of the Rat sarcoma (*Ras*)/mitogen‐activated protein kinase (*MAPK*) pathway.^39^, ^40^ *mTOR* is a critical downstream effector of the *PI3K/AKT* signaling pathway. Studies have demonstrated that certain miRNAs can modulate the activity of the *PI3K/AKT/mTOR* signaling pathway by directly targeting *mTOR*. *miR‐99a* and *miR‐144* have been shown to directly target *mTOR*, suppressing its expression. Nevertheless, their precise roles in RCC remain controversial and warrant further investigation for elucidation.^36^, ^41^ *PTEN* serves as a crucial negative regulator of the *PI3K/AKT/mTOR* signaling pathway. Numerous studies have demonstrated that miRNAs can indirectly modulate the activity of the *PI3K/AKT/mTOR* signaling pathway by targeting *PTEN*. These studies have revealed that miRNAs, including *miR‐23b*, miR‐193a‐3p, and *miR‐21*, can directly target *PTEN*, suppressing its expression, thus promoting the constitutive activation of the *PI3K/AKT/mTOR* signaling pathway and playing an oncogenic role in the initiation and progression of RCC.^36^ Moreover, certain miRNAs can modulate the activity of the *PI3K/AKT/mTOR* signaling pathway through alternative mechanisms. For instance, *miR‐193a‐3p* and *miR‐224* can modulate the activity of the *PI3K/AKT/mTOR* signaling pathway by targeting *ST3GalIV*.^42^ Concurrently, *miR‐124‐3p* can exert tumor‐suppressive effects by targeting *CAV1* and *FLOT1*, thus inhibiting the *PI3K/AKT* and *Ras/MAPK* signaling pathways.^43^

Endogenous molecules, such as fibroblast activation protein‐α (*FAP*) and cell division cycle‐associated 5 (*CDCA5*), have been demonstrated to promote the progression of RCC by activating the *PI3K/AKT* signaling pathway.^44^, ^45^ Knockdown of *FAP* expression attenuates the activity of the *PI3K/AKT/mTOR* signaling pathway, thereby inhibiting the growth of RCC, suggesting that targeting *FAP* may be a potential therapeutic strategy for RCC treatment.^44^ In contrast, high expression of *CDCA5* upregulates the *PI3K/AKT* signaling pathway, thereby stimulating RCC cell proliferation and invasion.^45^ Natural compounds, such as shikonin and thymoquinone (TQ), have also been shown to exert anti‐RCC effects by modulating the *PI3K/AKT* signaling pathway.^46^, ^47^ Shikonin inhibits RCC cell proliferation in a dose‐dependent manner and induces apoptosis by activating the *Ras/MAPK* and *PI3K/AKT* signaling pathways.^46^ Similarly, TQ inhibits RCC cell migration by suppressing the activation of the prostaglandin E2 (*PGE2*)‐mediated *EP2* receptor–*PI3K/AKT* axis.^47^

As a key downstream effector molecule of the *PI3K/AKT* signaling pathway, *mTOR* is commonly found in a state of sustained activation in RCC.^48^ Substantial evidence suggests that the *mTOR* signaling pathway is not only closely associated with the proliferation and differentiation of tumor cells but also plays a crucial role in the maintenance of cancer stem cells, potentially contributing to drug resistance in RCC.^48^, ^49^ In comparison with normal tissues, *mTOR* activity is significantly elevated in RCC tissues, and *mTOR* inhibitors have demonstrated efficacy in slowing the progression of RCC.^48^ Various mechanisms can lead to abnormal activation of *mTOR* in RCC, including *PTEN* functional deficiency, enhanced function of *PI3K* catalytic subunits, and *LKB1* gene mutations.^2^, ^50^, ^51^, ^52^, ^53^, ^54^, ^55^ Notably, *mTOR* activation primarily stems from an increase in its phosphorylation level rather than changes in protein expression, indicating that targeted inhibition of *mTOR* phosphorylation may be an important strategy for suppressing *mTOR* activity.^55^, ^56^

The *PI3K/AKT/mTOR* signaling pathway plays a pivotal role in the initiation and progression of renal cancer. Constitutive aberrant activation of this pathway exerts both direct and indirect promoting effects on renal cancer progression. Specifically, this pathway can directly promote tumor cell proliferation, survival, and invasion; furthermore, it can also indirectly facilitate renal cancer progression by maintaining cancer stem cells and conferring drug resistance. Consequently, drugs targeting *PI3K*, *AKT*, and *mTOR* could offer novel insights and therapeutic approaches for renal cancer treatment. Future research should focus on elucidating the molecular mechanisms underlying *PI3K/AKT/mTOR* pathway activation and identifying novel therapeutic targets to improve renal cancer diagnosis and treatment.

### 2.3 Hippo–YAP signaling pathway

The Hippo signaling pathway is a highly conserved signal transduction pathway throughout evolution, playing a crucial role in regulating organ size, maintaining tissue homeostasis, controlling cell proliferation, apoptosis, and other essential biological processes. This pathway consists of a series of kinase cascades, including the upstream mammalian Sterile 20‐like kinase 1/2 (*MST1/2*), large tumor suppressor 1/2 (*LATS1/2*) serine/threonine kinases, and the downstream transcriptional coactivators yes‐associated protein (*YAP*) and transcriptional coactivator with PDZ‐binding motif (*TAZ*). When the Hippo pathway is activated, *MST1/2* phosphorylates and activates *LATS1/2*, which in turn phosphorylates *YAP/TAZ*, leading to their cytoplasmic retention and degradation. Conversely, when the Hippo pathway is inactivated, nonphosphorylated *YAP/TAZ* translocate into the nucleus, binds to transcription factors such as the *TEAD* family, and promotes the transcriptional expression of downstream target genes, thereby promoting cell proliferation and inhibiting apoptosis. Accumulating evidence suggests that dysregulation of the Hippo–*YAP* pathway is closely associated with the occurrence and development of various tumors, including renal cancer.^57^

Numerous studies have demonstrated that aberrant activation of the Hippo signaling pathway is pervasive in renal cancer tissues.^57^ As an upstream regulatory gene of the Hippo pathway, mutations in the neurofibromatosis type 2 (*NF2*) gene can result in inactivation of the Hippo pathway and aberrant activation of *YAP*.^58^ Alterations in the Hippo pathway and persistent activation of *YAP* have been identified in *NF2*‐deficient uRCC.^57^, ^59^ Moreover, mechanistic studies have elucidated that silencing *YAP/TAZ* in *NF2*‐deficient tumors can facilitate tumor regression. The underlying mechanism is that the absence of *YAP/TAZ* enhances mitochondrial respiration, diminishes reliance on glycolytic growth, and results in the accumulation of reactive oxygen species (ROS) and oxidative stress‐induced cell death under nutrient‐deprived conditions.^57^, ^60^

The expression of the core kinases *LATS1/2* in the Hippo pathway is generally downregulated in RCC.^57^ Studies have demonstrated that RCC patients with high expression of *LATS1/2* exhibit significantly prolonged overall survival (OS) and disease‐free survival compared with those with low expression.^61^ Furthermore, elevated levels of methylation have been observed in the promoter region of *LATS1* in RCC tissues and cell lines. Treatment of RCC cells with the DNA methylation inhibitor 5‐aza‐2′‐deoxycytidine can demethylate *LATS1*, downregulate *YAP* expression, promote cell apoptosis, arrest the cell cycle, and inhibit cell proliferation.^62^ As key effector molecules downstream of the Hippo pathway, abnormal activation of *YAP/TAZ* is closely associated with the occurrence, progression, and prognosis of RCC.^57^ In ccRCC patients, *TAZ* expression was found to be significantly upregulated, and high expression of *TAZ* is indicative of a poor prognosis.^63^ Further studies have revealed that *TAZ* can induce the expression of *NADPH* oxidase 4 (*Nox4*) by regulating epithelial membrane protein 1 (*EMP1*). *Nox4* is enriched in the kidney and generates ROS associated with ferroptosis; therefore, the absence of *TAZ* can counteract ferroptosis.^57^, ^64^, ^65^

The Hippo–*YAP* pathway forms a complex cross‐regulatory network with multiple key molecules and signaling pathways in the occurrence and progression of RCC. Research suggests that the overexpression of transferrin and *B4GALNT1* may contribute to the occurrence and progression of ccRCC by modulating the Hippo pathway. Investigations have revealed that the downregulation of *SAV1* expression, a core component of the Hippo pathway, results in the aberrant activation of *YAP*, which plays a crucial role in the pathogenesis of high‐grade ccRCC.^57^, ^66^ Moreover, in mucinous tubular and spindle cell carcinoma (MTSCC), *YAP1* overexpression is a critical factor driving its pathogenesis, and pertinent studies have demonstrated that the *YAP* inhibitor verteporfin exhibits a potential therapeutic effect on metastatic MTSCC.^57^ Conversely, leukemia inhibitory factor receptor suppresses tumor metastasis by activating the Hippo pathway and downregulating YAP expression.^57^ Various noncoding RNAs (ncRNAs), including *TUG1* and *miR‐9*, have also been identified as regulators of renal cancer growth and metastasis through their modulation of *YAP* activity. Furthermore, molecules including Claudin‐2, *SH3BGRL2*, *QKI*, and REGγ/casein kinase 1ε (*CK1ε*) have also been demonstrated to exert critical regulatory functions in renal cancer progression through their interactions with the Hippo–YAP pathway. These research findings elucidate the intricate regulatory network of the Hippo–YAP pathway in renal cancer occurrence and progression, offering valuable insights for further elucidating the molecular mechanisms underlying renal cancer and identifying novel therapeutic targets.^57^

Overall, the dysregulation of the Hippo–*YAP* signaling pathway plays a pivotal role in the initiation and progression of renal cancer. Upstream regulators of the Hippo pathway, such as *NF2* and *LATS1/2*, are frequently mutated or downregulated in renal cancer tissues, resulting in pathway inactivation. Conversely, downstream effector molecules, including *YAP/TAZ*, are aberrantly activated, promoting renal cancer cell proliferation and survival. The Hippo–*YAP* pathway has an extensive cross‐regulatory network with multiple critical molecules and signaling cascades, which collectively participate in regulating renal cancer progression. Comprehensive characterization of the dysregulation mechanisms of the Hippo–*YAP* pathway is anticipated to provide novel insights and strategies for targeted therapy of renal cancer. Interventions targeting pivotal components of this pathway, including *LATS1/2* and *YAP/TAZ*, may serve as effective approaches for the future treatment of renal cancer.

### 2.4 Wnt/β‐catenin signaling pathway

The *WNT*/β‐catenin pathway is a highly conserved signal transduction pathway that plays a pivotal role in various biological processes, including embryonic development, organogenesis, and maintenance of tissue homeostasis. This pathway primarily consists of *Wnt* ligands, transmembrane receptors, cytoplasmic protein degradation complexes, and nuclear transcription factors. In the absence of *Wnt* ligand stimulation, cytoplasmic β‐catenin is phosphorylated by kinases such as glycogen synthase kinase 3β (GSK3β), which subsequently leads to its ubiquitination and degradation, thus maintaining low levels of β‐catenin. Upon *Wnt* ligand binding to transmembrane receptors, GSK3β kinase activity is suppressed, resulting in the accumulation of β‐catenin in the cytoplasm and its subsequent translocation into the nucleus. In the nucleus, β‐catenin interacts with transcription factors such as *TCF*/*LEF*, activating the transcription of downstream target genes and ultimately triggering a cascade of cell fate determination events. In addition to the canonical *Wnt*/β‐catenin pathway, the noncanonical *Wnt*/Ca2+ and Wnt/planar cell polarity pathways also play crucial roles in regulating cell polarity and migration.^19^

In recent years, numerous studies have demonstrated that the *WNT*/β‐catenin signaling pathway is frequently aberrantly activated during the development of various solid tumors and is closely associated with the malignant phenotype of these neoplasms.^67^, ^68^, ^69^ In RCC, the key components of the *Wnt*/β‐catenin signaling pathway are generally dysregulated.^19^ For instance, canonical *Wnt* ligands, such as *Wnt1* and *Wnt10A*, are highly expressed in RCC tissues and are associated with tumor staging and invasiveness.^70^, ^71^ Conversely, noncanonical ligands, including *Wnt7A* and *Wnt5A*, are downregulated,^72^, ^73^ and their reduced expression may also be implicated in the development of renal cancer.^19^ Notably, β‐catenin frequently accumulates aberrantly in RCC tissues, and its cytoplasmic levels are closely correlated with tumor diameter, staging, and prognosis.^74^, ^75^ Although β‐catenin gene mutations are relatively uncommon in RCC, multiomics analyses have confirmed that genes associated with the *WNT*/β‐catenin pathway generally undergo genetic and epigenetic alterations.^19^ In addition to *Wnt* ligands and receptors, antagonists such as secreted frizzled‐related proteins (*sFRPs*), *WNT* inhibitory factor 1, and Dickkopf (*DKK*) family members are also frequently deficient in RCC tissues, further exacerbating the sustained activation of the *WNT*/β‐catenin signaling pathway.^19^ The overexpression of genes such as *CENPA* and *GBP2* can accelerate the cell cycle progression of RCC cells by activating the *WNT*/β‐catenin pathway, thereby promoting their proliferation, migration, and invasion.^76^, ^77^ Moreover, the sustained activation of the *WNT*/β‐catenin pathway can lead to chemotherapy drug tolerance in RCC cells and induce T cell differentiation into stem cell memory T‐cell subpopulations, thus enhancing the tumor's immune escape capability.^78^ These results suggest that the dysregulated expression of *WNT*/β‐catenin pathway components may be a crucial factor driving the occurrence and development of RCC, and targeting this pathway could potentially serve as an effective strategy for RCC treatment.

The *WNT*/β‐catenin pathway does not function independently during the development of RCC; instead, it is closely interconnected with multiple other signaling pathways. Research has demonstrated that the *WNT*/β‐catenin pathway can promote carcinogenesis by modulating the expression of oncogenes and cell cycle regulators, including *c‐Myc* and Cyclin D1.^19^ Moreover, the *WNT*/β‐catenin pathway can act synergistically with the *VHL–HIF* pathway to augment the motility and invasive potential of RCC cells. Nonetheless, *HIF‐1α* and *HIF‐2α* appear to exert opposing biological effects in RCC,^19^ and the maintenance of intracellular homeostasis following *VHL* inactivation is contingent upon the delicate balance between these *HIF* isoforms.^79^, ^80^ Consequently, further investigation is necessary to elucidate the interaction patterns among the *VHL*, *HIF*, and *WNT*/β‐catenin pathways and their influence on the RCC phenotype. Recent studies have also revealed that multiple miRNAs can function as either oncogenes or tumor suppressor genes in the progression of RCC by targeting various components of the *WNT*/β‐catenin signaling pathway.^36^ For instance, *miR‐106b‐5p*, *miR‐1260b*, and *miR‐203a* target the negative regulatory factors *LZTFL1*, *SFRP1*, *DKK2*, and *GSK3β* of the *WNT*/β‐catenin pathway, respectively, thereby promoting RCC cell proliferation, invasion, and stem cell phenotype, and thus acting as oncogenes.^81^, ^82^, ^83^ Conversely, *miR‐372* targets the *IGF2BP1* gene, inhibiting the *WNT*/β‐catenin pathway and exhibiting tumor suppressor properties.^84^ These research findings unveil the intricate regulatory network of the *WNT*/β‐catenin signaling pathway in the development and progression of RCC, offering crucial insights into the molecular mechanisms underlying this malignancy. Future research should focus on elucidating the crosstalk between the *WNT*/β‐catenin signaling pathway and other signaling cascades, as well as unraveling the precise mechanisms by which various miRNAs regulate this pathway. Such endeavors will facilitate the identification of novel therapeutic targets and strategies for RCC management.

The function of noncanonical *Wnt* signaling pathways in RCC has not been extensively studied, and the related research is relatively limited compared with that of the *Wnt*/β‐catenin signaling pathway.^19^ Receptor tyrosine kinase (RTK)‐like orphan receptor 2 (*RoR2*) is a type of *Wnt* ligand receptor that is typically expressed only during embryonic development. However, it is overexpressed in ccRCC.^85^ Suppressing the expression of *Ror2* in RCC using shRNA or mutagenesis methods can effectively attenuate tumor growth, cell migration, and invasion promoted by *Wnt/Rho* signal transduction.^85^, ^86^ The activity of DICKKOPF‐3 (*Dkk‐3*), an antagonist of the *Wnt* pathway, is reduced in RCC. Restoring its expression can not only inhibit tumor cell proliferation but also promote cell apoptosis.^87^

In summary, the *WNT*/β‐catenin signaling pathway plays a pivotal role in the initiation and progression of renal cancer. On one hand, components of the *Wnt*/β‐catenin signaling pathway, including *Wnt* ligands, receptors, and β‐catenin, are frequently dysregulated in renal cancer tissues. Furthermore, the loss of *Wnt* antagonists exacerbates the constitutive activation of the pathway. On the other hand, the aberrantly activated *Wnt*/β‐catenin signaling pathway confers multiple malignant phenotypes upon renal cancer cells, including enhanced proliferation, invasion, drug resistance, and immune evasion, by regulating downstream target genes. Moreover, this pathway synergistically promotes renal cancer progression in cooperation with other signaling pathways, such as the *VHL–HIF* pathway, while also being intricately regulated by ncRNAs, including miRNAs. These research findings not only enhance our understanding of renal cancer pathogenesis but also provide a theoretical basis for the development of targeted therapies against the *WNT*/β‐catenin signaling pathway. It is anticipated that as the regulatory network and aberrant activation mechanisms of the *WNT*/β‐catenin signaling pathway in tumors are further elucidated, novel therapeutic strategies will continue to emerge, ultimately benefiting a substantial number of renal cancer patients.

### 2.5 cAMP signaling pathway

cAMP is a pivotal second messenger molecule that plays a crucial role in intracellular signaling. It is primarily synthesized by membrane‐bound adenylyl cyclase through the catalysis of adenosine triphosphate (ATP) and is subsequently hydrolyzed by phosphodiesterase into adenosine monophosphate (AMP). cAMP is involved in the regulation of various cellular functions, including cell growth, differentiation, apoptosis, and metabolism. cAMP primarily binds to effector molecules, such as protein kinase A (*PKA*) and cyclic AMP‐responsive element‐binding protein (*CREB*), thereby regulating downstream signaling pathways and gene expression.^2^ In recent years, numerous studies have demonstrated that the cAMP signaling pathway plays a significant role in the development and progression of various tumors; however, its specific mechanisms in renal cancer have not been fully elucidated and warrant further in‐depth investigation.

Studies have discovered that increased synthesis of acetylcholine (ACh) activates the *cAMP/PKA* pathway through its receptors, subsequently phosphorylating *CREB*, leading to invasive migration and proliferation of renal cancer cells.^88^, ^89^, ^90^ Phosphorylated *CREB* can promote the invasive metastasis of renal cancer by regulating the expression of matrix metallopeptidases (*MMP2* and *MMP9*) and EMT‐related proteins.^89^ Moreover, research indicates that the level of *CREB* phosphorylation is upregulated in ccRCC tissues and cell lines, while inhibiting *CREB* phosphorylation at the serine 133 site can significantly suppress the growth and metastatic activity of OS‐RC‐2 cells.^88^, ^89^ In addition to ACh, dysregulation of the cAMP signaling pathway may be associated with the development of renal cancer. An analysis of ccRCC cell lines confirmed the abnormal expression of *CREB1* protein, suggesting that a posttranscriptional regulatory mechanism may contribute to *CREB1* dysregulation.^91^ As one of the downstream effector molecules of cAMP, CREB activity is regulated by cAMP levels. Under the influence of the tumor microenvironment (TME), the cAMP signaling pathway can exert a dual effect on tumor cell growth, either promoting or inhibiting it.

In summary, the cAMP signaling pathway is implicated in the regulation of renal cancer initiation and progression through diverse mechanisms. On one hand, factors such as ACh can activate the *cAMP/PKA/CREB* pathway, leading to the upregulation of *MMP2/9*) and EMT‐related molecules, thereby promoting the proliferation, invasion, and metastasis of renal cancer cells. On the other hand, the dysregulation of cAMP signaling molecules, particularly *CREB*, is strongly associated with renal cancer, indicating that targeting the cAMP pathway and its downstream effectors may offer novel strategies for the prevention and treatment of this malignancy. In future studies, it will be crucial to further elucidate the molecular mechanisms underlying the cAMP signaling pathway's role in regulating the initiation and progression of renal cancer, facilitating the identification of potential therapeutic targets.

### 2.6 HGF/c‐Met signaling pathway

*c‐Met*, a transmembrane tyrosine kinase receptor, is activated by *HGF* and plays a crucial role in regulating cell migration under both physiological and pathological conditions. Extensive research has shown that c‐Met is implicated in the EMT process of multiple cancer types. Moreover, the interaction between *c‐Met* and *HGF* can stimulate tumor cell mitosis, motility, angiogenesis, migration, and invasion.^92^ Recently, the oncogenic role of *c‐Met* in urinary system malignancies, particularly in renal cancer, has garnered significant attention.

*VHL* gene mutations and hypoxic conditions can lead to the upregulation of *HGF* and its receptor *c‐Met* in renal cancer.^93^, ^94^, ^95^ Furthermore, *HIF‐1* can also regulate the expression of *c‐Met* and *VEGF* under hypoxic conditions.^96^ These findings suggest that the *HGF*/*c‐Met* signaling pathway may contribute to the progression of renal cancer by promoting tumor angiogenesis. Consequently, *c‐Met* has emerged as a crucial target for antiangiogenic therapy in renal cancer. Besides inducing tumor angiogenesis, *c‐Met* can also facilitate the malignant progression of renal cancer through alternative mechanisms. Research has demonstrated that the phosphorylation level of the *c‐Met* receptor positively correlates with tumor growth, vascularization, propensity for lung metastasis, and cell migration capacity in renal cancer.^97^ Moreover, an increase in *MET* gene copy number is also linked to poor prognosis and distant metastasis in patients with ccRCC.^98^ These findings further highlight the crucial role of *c‐Met* in the metastatic cascade of renal cancer.

Given the crucial role of the *HGF/c‐Met* pathway in the pathogenesis and progression of RCC, therapeutic strategies targeting this pathway have emerged as a promising area of research. For example, the combination of axitinib, a *VEGF* inhibitor, and crizotinib, a *c‐Met* inhibitor, has demonstrated significant improvements in the therapeutic efficacy of RCC by simultaneously targeting these two key pathways.^92^ Furthermore, natural small‐molecule compounds, including honokiol, rapamycin, and piperine, have exhibited anti‐RCC effects by inhibiting *c‐Met* activity, thus demonstrating promising potential for clinical application.^99^

In summary, the *HGF/c‐Met* signaling pathway plays a pivotal role in the initiation and progression of renal cancer. First, this pathway can directly promote renal cancer progression by facilitating tumor angiogenesis and EMT. Second, abnormalities in *c‐Met* activity and *MET* gene copy number are also strongly correlated with renal cancer metastasis. Consequently, therapeutic strategies targeting the *HGF/c‐Met* axis hold promise for providing novel treatment options for patients with advanced and metastatic renal cancer. Future research should further elucidate the molecular mechanisms underpinning the role of the *HGF/c‐Met* pathway in renal cancer initiation and progression and explore safer and more effective c‐Met inhibitors, ultimately providing innovative strategies for the precision treatment of renal cancer.

### 2.7 p53 signaling pathway

In the context of renal cancer, the *p53* signaling pathway plays a critical role. The *p53* protein, encoded by the *TP53* gene, is a crucial tumor suppressor that maintains genomic integrity and inhibits tumor development. Under physiological conditions, *p53* is activated in response to various stress signals, initiating downstream transcriptional programs that suppress tumor growth. Upon detection of cellular damage or stress, *p53* triggers various biological processes, including cell cycle arrest, DNA repair, apoptosis, and autophagy, which maintain genomic stability and prevent malignant cell transformation.^2^, ^9^

The dysregulation of the *p53* signaling pathway is closely associated with the initiation and progression of RCC.^9^ Studies have demonstrated that although the mutation rate of the *TP53* gene in RCC is relatively low, the loss of *p53* activity remains a common characteristic of this malignancy.^9^, ^100^, ^101^ This finding suggests that alternative mechanisms may exist that impede the transmission of *p53* signaling in RCC. For instance, the activation of *mTOR* and the inactivation of the *p38MAPK‐p53/p16* pathway are believed to synergistically trigger the transformation of the proximal renal tubule into RCC.^102^ Moreover, the interplay between *p53* and the *VHL* gene is essential for regulating *p53*‐mediated DNA damage response, and their dysregulation may facilitate the progression of RCC.^103^ Additionally, there exists an intricate regulatory relationship between *p53* and *HIF‐1α*. Certain studies propose that *HIF‐1α* can induce the inhibition of *MDM2*, thus stabilizing *p53* and promoting cellular apoptosis^104^, ^105^; conversely, other investigations suggest that hypoxia and HIF‐1α may negatively regulate the stability and activity of *p53* under specific conditions.^104^ These findings underscore the extensive cross‐talk between the *p53* pathway and other critical pathways implicated in the pathogenesis and progression of RCC.

In addition to *p53* itself, other components of the *p53* pathway are also implicated in the pathogenesis of renal cancer. *PPM1D*, a transcriptional target gene of *p53*, can reverse DNA damage checkpoints and attenuate cell cycle arrest by dephosphorylating *p53* and *Chk1*.^106^ *MDM2*, a negative regulator of *p53*, promotes the degradation of *p53* through ubiquitin‐mediated proteasomal degradation.^107^ In addition to *MDM2*, other molecules, including *ARF‐BP1*/Mule, *MdmX/Mdm4*, *Cop1*, and *Pirh2*, have been identified as negative regulators of *p53* signaling.^2^, ^108^ Alterations in these factors that regulate the stability and activity of *p53* are likely to contribute to the loss of *p53* function in RCC.

In summary, the *p53* signaling pathway plays a pivotal role in the initiation and progression of renal cancer. On one hand, as the guardian of the genome, the loss of *p53* activity renders renal cells vulnerable to malignant transformation; on the other hand, there exists significant reciprocal regulation between the *p53* pathway and critical renal cancer pathways such as *VHL* and *HIF*, and the imbalance among these pathways further facilitates tumor progression. In‐depth investigation of *p53* pathway abnormalities not only contributes to elucidating the molecular mechanisms underlying renal cancer but also provides new insights and potential therapeutic targets for the diagnosis and treatment of this malignancy. Moving forward, it is imperative to further elucidate the mechanisms by which *p53* signaling integrates various stress responses and investigate strategies for targeted regulation of the *p53* pathway, thereby providing more clues for precise diagnosis and personalized treatment of renal cancer.

### 2.8 Ferroptosis‐related signaling pathways

Ferroptosis, an emerging form of regulated cell death, is characterized by iron‐mediated lipid peroxidation on the cell membrane, distinguishing it from other types of cell death, such as apoptosis and necroptosis, in terms of morphological and molecular mechanisms. The primary characteristic of ferroptotic cells is the shrinkage of mitochondrial cristae, without displaying typical apoptotic features, including chromatin condensation and apoptotic body formation. The occurrence of ferroptosis depends on three crucial factors: (1) the synthesis of polyunsaturated fatty acid phospholipids (*PLs*) and their iron‐catalyzed peroxidation; (2) the regulation of iron metabolism, including the promotion of the Fenton reaction and serving as an essential cofactor for lipid peroxidases; and (3) the modulation of mitochondrial metabolism, as mitochondria facilitate ferroptosis through their roles in bioenergetics, biosynthesis, and ROS generation. This iron‐dependent PL peroxidation process is stringently regulated by various intracellular metabolic pathways, encompassing redox balance, iron metabolism, mitochondrial activity, amino acid and lipid metabolism, and glucose metabolism. It is crucial to highlight the pivotal role of iron metabolism in the regulatory network governing ferroptosis.^109^

Recent investigations have demonstrated that ferroptosis is suppressed during the initiation and progression of renal cancer. Elucidating the mechanisms by which RCC tumors inhibit cellular ferroptosis and developing strategies to reactivate ferroptosis may offer novel insights for RCC treatment. ccRCC, the predominant subtype of RCC, exhibits a distinct metabolic profile that is intimately associated with its sensitivity to ferroptosis. Protein disulfide‐isomerase A4 (*PDIA4*) confers resistance to ferroptosis in ccRCC cells by upregulating *ATF4/SLC7A11*, whereas salinomycin exerts antitumor effects by suppressing PDIA4.^110^ Moreover, Glutathione peroxidase 4 (*GPX4*) plays a pivotal role in ccRCC progression, and Kruppel Like Factor 2 (*KLF2*) is implicated in the regulation of *GPX4* expression in ccRCC. Overexpression of *KLF2* suppresses tumor growth and invasion by modulating ferroptosis.^111^ Acyl CoA long‐chain synthetase 3 (*ACSL3*) regulates lipid droplet accumulation in ccRCC, a process crucial for tumor growth, and also modulates ferroptosis sensitivity in a manner dependent on the composition of exogenous fatty acids. Both ferroptosis‐inducing and ferroptosis‐inhibiting functions can be exploited for the treatment of clear ccRCC. The absence of *AIM2*, a tumor suppressor, promotes ccRCC progression and sunitinib resistance through the inhibition of ferroptosis regulated by the *FOXO3a/ACSL4* axis.^112^ Similarly, the Hippo pathway effector *TAZ* and iron–sulfur cluster assembly enzyme 2 (*ISCA2*) have been shown to induce ferroptosis in ccRCC.^113^ Furthermore, a prognostic model based on eight ferroptosis‐related long noncoding RNAs (lncRNAs) has been developed for predicting the prognosis of ccRCC patients. The combination of URB597, a fatty acid amide hydrolase (*FAAH*) inhibitor, and (1S,3R)‐RSL3, a ferroptosis inducer, demonstrates potent synergistic inhibition of RCC growth by inducing G1 cell cycle arrest and promoting ROS generation. This dual‐targeted therapy modulates the sensitivity of RCC cells to ferroptosis.^114^

In conclusion, ferroptosis plays a pivotal role in the initiation and progression of renal cancer. The distinct metabolic profile of renal cancer is intimately associated with its sensitivity to ferroptosis, with multiple molecular mechanisms implicated in the regulation of ferroptosis in renal cancer, including *PDIA4*, *GPX4*, *KLF2*, *ACSL3*, and *AIM2*. Ferroptosis holds promise as a novel therapeutic target and offers potential avenues for the prevention, diagnosis, and treatment of renal cancer.

### 2.9 Cuproptosis‐related signaling pathways

Copper is one of the essential trace elements that maintains the balance of the internal environment in the human body. As a cofactor of various enzymes, copper participates in regulating multiple physiological processes within cells, such as the function of cytochrome coxidase (*COX*), which requires the involvement of copper ions to complete cellular respiration.^115^ However, recent research indicates that excessive intracellular copper concentrations can trigger a unique form of cell death known as “cuproptosis.”^116^ The primary mechanism of cuproptosis involves the binding of excess copper to succinylated proteins in the tricarboxylic acid cycle, leading to abnormal aggregation of lipidation‐related proteins and degradation of iron–sulfur cluster proteins, ultimately resulting in cell death due to an intracellular protein toxicity stress response.^116^ Appropriate levels of copper can induce autophagy‐mediated degradation of *GPX4*, thereby promoting ferroptosis, indicating a crosstalk between ferroptosis and cuproptosis.^117^

ccRCC is insensitive to conventional radiotherapy and chemotherapy, which may be attributed to its resistance to cell death‐related signaling pathways. Currently, various forms of cell death, such as apoptosis, necrosis, pyroptosis, ferroptosis, and autophagic cell death, have been extensively studied in ccRCC^22^; however, the role of cuproptosis in the pathogenesis and progression of ccRCC remains largely unexplored.^115^ Multiple studies have demonstrated that copper levels are elevated to varying degrees in ccRCC patients, suggesting the potential involvement of cuproptosis in the pathogenesis and progression of ccRCC. For instance, Pirincci et al.^118^ reported that serum copper levels were significantly higher in ccRCC patients compared with the control group. Similarly, Panaiyadiyan et al.^119^ observed a significant elevation in blood copper concentration in renal cancer patients. Furthermore, elevated expression of the copper transporter protein CTR2 is associated with poor prognosis in ccRCC.^120^

Cuproptosis may contribute to metabolic reprogramming in ccRCC by modulating the activity of crucial mitochondrial enzymes.^115^ Dysregulation of the *VHL/HIF* pathway and perturbations in copper homeostasis may synergistically contribute to metabolic reprogramming in ccRCC, offering a novel insight into the role of cuproptosis in the initiation and progression of ccRCC. Mitochondrial dysfunction and metabolic reprogramming are hallmark features of ccRCC.^121^ Deletion of the *VHL* gene and aberrant activation of the *HIF* pathway are pivotal in driving the metabolic reprogramming of ccRCC.^115^ Deletion of the *VHL* gene results in enhanced stability of *HIF‐1α* and *HIF‐2α* proteins, leading to upregulated glycolysis and consequently diminished mitochondrial respiration and energy metabolism.^115^ Subsequent investigations have revealed an intricate regulatory interplay between copper and the *HIF‐1* pathway.^115^ On the one hand, copper can enhance the expression and transcriptional activity of *HIF‐1*, thereby promoting the expression of its downstream target genes, such as *VEGF*.^122^, ^123^ Conversely, *HIF‐1* can also negatively modulate the expression of copper transport proteins via posttranscriptional mechanisms, such as suppressing the expression of copper transporter 1A (*CTR1A*).^124^ ATPase copper‐transporting beta (*ATP7B*) is a copper‐transporting ATPase that exhibits markedly increased expression in ccRCC, potentially serving as a compensatory mechanism in response to elevated copper levels.^125^

Another important characteristic of cuproptosis is the disruption of lipoic acid metabolism and Fe–S cluster proteins.^115^ Studies have demonstrated that the use of copper ion carriers leads to the depletion of Fe–S cluster proteins in an *FDX1*‐dependent manner, thereby triggering proteotoxic stress.^116^ In ccRCC, the expression of multiple Fe–S cluster proteins, such as *FDX1*, *LIAS*, *ACO‐2*, and *SDHB*, is generally reduced and is associated with a poor prognosis.^115^ Furthermore, *FDX1* knockout can suppress cuproptosis in ccRCC cells.^126^ Research has also revealed that the expression and activity of key enzymes involved in lipoic acid biosynthesis, such as *LIAS* and *LIPT*, are altered in ccRCC cells, leading to the dysfunction of lipoylated proteins, which may contribute to metabolic reprogramming in ccRCC.^115^, ^127^ It is worth noting that the activation of the *HIF* pathway induced by *VHL* gene deletion can suppress the expression of the Fe–S protein *SDHD* by upregulating miR‐210, suggesting that the *VHL/HIF* pathway is implicated in cuproptosis regulation.^115^

In summary, cuproptosis, a recently discovered form of cell death, is strongly correlated with the onset and advancement of renal cancer. Patients with renal cancer frequently exhibit elevated copper levels. Furthermore, cuproptosis‐associated molecular mechanisms, including mitochondrial dysfunction, Fe–S cluster protein dysregulation, and protein persulfidation, might contribute to the metabolic reprogramming observed in renal cancer.

### 2.10 NF‐κB signaling pathway

*NF‐κB* is a ubiquitous transcription factor in mammalian cells, generally existing as heterodimers.^128^ The canonical *NF‐κB* pathway involves complexes formed by p50 and RelA, while the noncanonical *NF‐κB* pathway is primarily associated with p52/RelB.^128^ The *NF‐κB* pathway is involved in the inflammatory stress response triggered by cytokines, bacterial toxins, viral products, and cell death stimuli.^129^ Under physiological conditions, the *NF‐κB* pathway regulates the innate immune system, whereas its abnormal activation may lead to pathological reactions during tumor development.^130^ For example, the *NF‐κB* pathway can promote cancer cell migration and invasion by upregulating multidrug resistance genes, proangiogenic factors, and proinflammatory cytokines, such as *EGF*, *VEGFA*, *IL‐8*, and *IL‐6*.^131^ The *NF‐κB* subunit p50 can regulate the transition of macrophages from a protumorigenic phenotype to an M1 type, thereby inhibiting tumor growth.^132^ *VHL* deficiency can activate the *NF‐κB* pathway,^133^ which in turn induces the expression of antiapoptotic genes *Bcl‐xL* and *Bcl‐2* and suppresses the expression of tumor suppressor genes (such as *p53*), suggesting its potential role in the development of renal cancer.^129^, ^134^ Increased expression of *NF‐κB* is associated with lower survival rates in RCC patients.^135^ Activation of the *NF‐κB* and *STAT3* pathways increases the infiltration of regulatory T cells (Tregs) in tumor tissues, thereby promoting the initiation, development, and metastasis of renal cancer.^129^, ^136^

### 2.11 TGF‐β signaling pathway

*TGF‐β* is a class of evolutionarily highly conserved, multifunctional cytokines that play crucial roles in regulating various biological processes, including cell proliferation, differentiation, apoptosis, and the synthesis and degradation of extracellular matrix (ECM).^137^, ^138^ The TGF‐β family consists of three subtypes: *TGF‐β1*, *TGF‐β2*, and *TGF‐β3*, with *TGF‐β1* being abundantly expressed in the kidneys. TGF‐β exerts its effects by binding to its specific receptors, namely type I and type II transmembrane serine/threonine kinase receptors. Upon ligand binding, the type II receptor recruits and phosphorylates the type I receptor, which in turn is activated and specifically phosphorylates the intracellular R‐Smad proteins (*Smad2/3*).^138^ Phosphorylated R‐Smads form heterotrimeric complexes with Co‐Smad (*Smad4*), translocate into the nucleus, and interact with other transcription factors, coactivators, or corepressors to regulate the transcriptional expression of downstream target genes.^12^

In adult kidneys, the *TGF‐β/Smad* signaling pathway plays a crucial role in regulating the pathophysiological processes of glomeruli and renal tubules.^139^, ^140^ Numerous studies have demonstrated that aberrant activation of TGF‐β1 is strongly correlated with the development of renal fibrosis. TGF‐β1 can stimulate the synthesis of ECM proteins, including fibronectin and type I collagen, while suppressing the expression of MMPs. This imbalance leads to an excessive accumulation of ECM, ultimately resulting in renal interstitial fibrosis and loss of renal function.^2^, ^139^ Apart from its involvement in the process of renal fibrosis, the TGF‐β signaling pathway is also intimately associated with the initiation and progression of renal cancer.^12^, ^141^ Accumulating evidence suggests that TGF‐β plays a paradoxical role in the progression of renal cancer.^142^, ^143^, ^144^ In the early stages of renal cancer, TGF‐β acts as a tumor suppressor by inhibiting cell proliferation and promoting cell apoptosis. However, as the renal tumor advances, cancer cells progressively become resistant to TGF‐β‐mediated growth inhibition and apoptosis. Instead, they acquire phenotypic changes that facilitate tumor invasion and metastasis.^12^, ^145^, ^146^, ^147^, ^148^, ^149^, ^150^, ^151^, ^152^

Mechanistic studies have demonstrated that TGF‐β promotes the malignant progression of RCC through multiple signaling pathways. TGF‐β1 can promote the invasion and metastasis of RCC cells by regulating the expression of secreted protein acidic and rich in cysteine (SPARC), activating the AKT pathway, and upregulating the expression of MMP2.^153^ Furthermore, TGF‐β can also exert a protumorigenic effect by regulating the expression of ncRNAs. For instance, the overexpression of TGF‐β1‐induced long noncoding RNA‐activated by TGF‐β (lncRNA‐ATB) and SPRY4 intronic transcript 1 (SPRY4‐IT1) can promote EMT and the invasive capacity of RCC cells. Conversely, inhibiting TGF‐β‐induced EMT can effectively suppress the metastasis of RCC.^154^, ^155^ Moreover, there exists a cross‐regulation between the TGF‐β signaling pathway and the hypoxia pathway caused by VHL gene deletion.^152^ Studies have revealed that VHL gene knockout can activate the TGF‐β pathway, promoting the proliferation and survival of RCC cells. Conversely, inhibiting TGF‐β can attenuate the invasive capacity of ccRCC cells caused by VHL deficiency.^12^ These findings suggest that targeting the TGF‐β signaling pathway may provide novel therapeutic strategies for the treatment of RCC.

In summary, the TGF‐β/Smad signaling pathway plays a pivotal role in the pathophysiological processes of the kidney and the progression of renal cancer. TGF‐β facilitates the onset and progression of renal fibrosis by inducing the accumulation of ECM proteins. During the progression of renal cancer, TGF‐β exhibits a dual role, exerting antitumor effects in the early stages, while promoting EMT, invasion, and metastasis of tumor cells in the advanced stages. Elucidating the molecular mechanisms of TGF‐β in the development of kidney diseases and renal cancer will facilitate the identification of novel diagnostic biomarkers and therapeutic targets, offering new perspectives for the prevention and treatment of renal cancer.

## 3 TREATMENT IN RENAL CANCER

**FIGURE 3:**
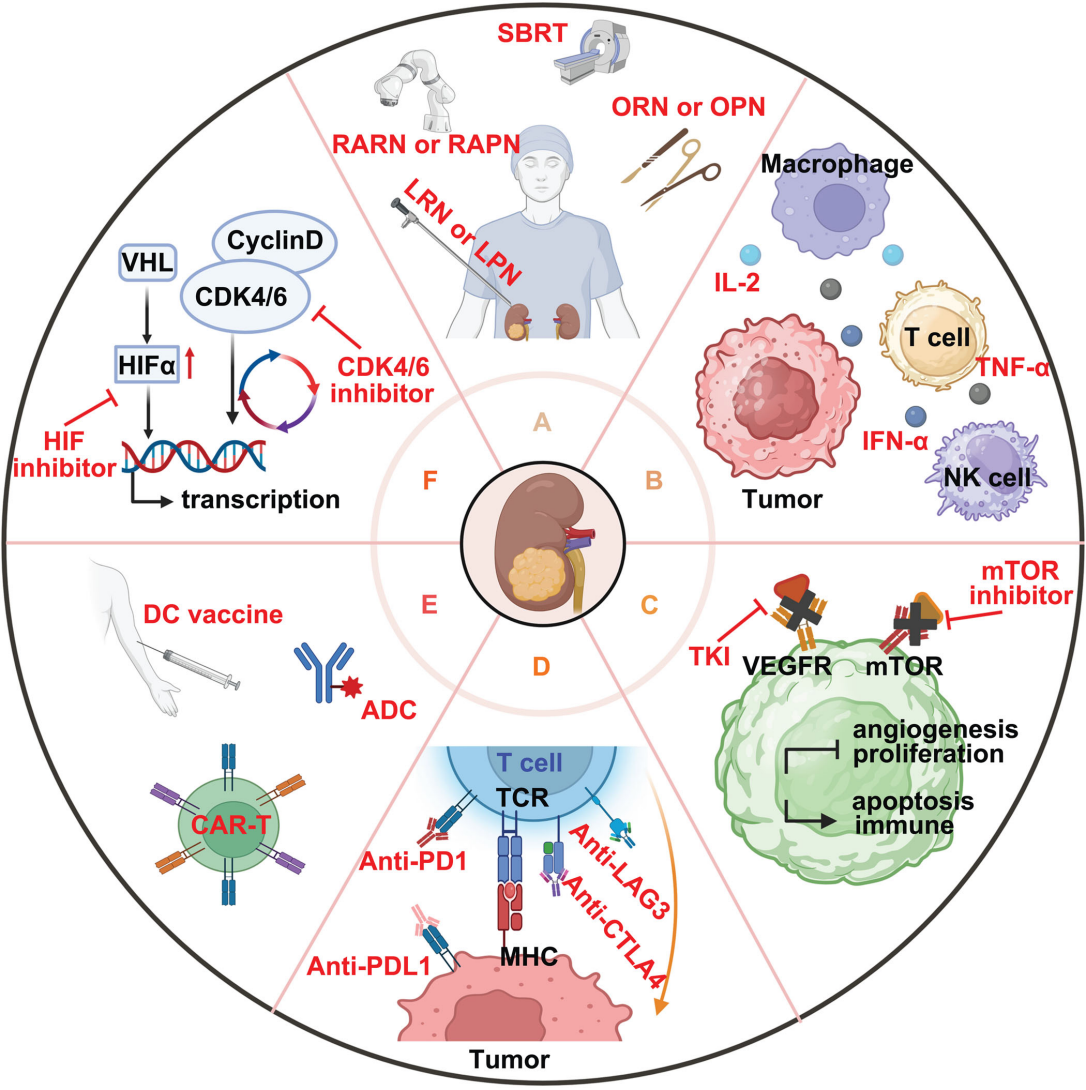
The therapeutic strategies in renal cancer. (A) Major surgical treatment options and SBRT for renal cancer. OPN, open partial nephrectomy; ORN, open radical nephrectomy; RAPN, robot‐assisted partial nephrectomy; RARN, robot‐assisted radical nephrectomy; LPN, laparoscopic partial nephrectomy; LRN, laparoscopic radical nephrectomy. (B) Cytokine therapy options for renal cancer, including IL‐2, TNF‐α, and IFN‐α. (C) Targeted therapy options for renal cancer, including TKIs and mTOR inhibitors. (D) Immune checkpoint inhibitor options for renal cancer, including anti‐PD‐1, anti‐PD‐L1, anti‐LAG3, and anti‐CTLA4. (E) Novel immunotherapy options for renal cancer, including DC vaccines, ADC, and CAR‐T. (F) Novel targeted therapy options for renal cancer, including HIF inhibitors and CDK4/6 inhibitors. This figure was created based on the tools provided by Biorender.com.

Renal cancer is considered a tumor type that is insensitive to both radiation^156^ and chemotherapy.^157^ For local lesions, surgical treatment remains the first choice.^158^ However, for advanced renal cancer, systematic and comprehensive treatment is often required.^159^ The treatment of advanced and metastatic renal cancer has evolved through stages such as cytokine therapy, targeted therapy, and more recently, the application of immunotherapy. ICIs are now used in treating various malignant tumors, including renal cancer.^160^ Additionally, new therapeutic targets are continually being discovered, leading to new renal cancer treatments such as chimeric antigen receptor (CAR)‐T cell therapy, dendritic cells (DCs) vaccine, cyclin‐dependent kinase (CDK) and *HIF* inhibitor, antibody–drug conjugates (ADCs), and the research of stereotactic body radiation therapy (SBRT) and gut microbiota (Figure 3).

### 3.1 Surgical treatment

Surgical resection is irreplaceable in treating localized RCC, making it the preferred option. Commonly used nephrectomy modalities include radical nephrectomy (RN) and partial nephrectomy (PN). RN implies the removal of the entire kidney, perirenal adipose tissue, and Gerota's fascia, maximizing the resection of the diseased area. However, RN requires a large incision, especially in open RN, leading to acute nephron loss, which increases the load on the contralateral kidney and risks postoperative acute kidney injury.^161^ PN, including nephron‐sparing surgery (NSS), removes only the lesion site, preserving as many normal nephrons as possible, thus better preserving postoperative renal function and reducing cardiovascular disease risk.^162^ In early‐stage tumor lesions, NSS presents noninferiority compared with RN. Although PN can lead to complications like hematuria, perinephric hematoma, and urinary fistulae, it is recommended for early‐stage RCC patients, especially T1 and T2, when technically feasible.^163^ Recent studies indicate that removing the primary tumor in metastatic RCC can benefit patients through cytoreductive nephrectomy (CN) or debulking nephrectomy.^164^ CN works synergistically with immunotherapy by eliminating the primary tumor and removing cytokines and proteins that inhibit the immune response.^165^ Studies comparing CN combined with *IFN‐α* to IFN‐α alone in mRCC patients show that combination therapy significantly improves median survival (13.6 vs. 7.8 months), regardless of performance status, metastasis site, and measurable disease presence.^166^ However, with the advent of targeted therapies and immunotherapy, the benefits of CN in comprehensive mRCC treatment remain controversial for both immediate and deferred CN.^167^ More studies are needed to confirm CN's value and role.

Minimally invasive surgery (MIS), including laparoscopic and robotic‐assisted surgery, has significant OS advantages over open surgery for stage I and II RCC. MIS also has lower readmission rates (2.4 vs. 2.87%), 30‐day mortality rates (0.53 vs. 0.96%), and 90‐day mortality rates (1.04 vs. 1.77%).^168^ For T3 stage RCC, the laparoscopic radical nephrectomy (LRN) group had lower estimated blood loss (100 vs. 650 mL, *p* < 0.001) and shorter hospital stays (4 vs. 9 days, *p* < 0.001) compared with the open radical nephrectomy (ORN) group. This suggests that LRN performs better than ORN in the perioperative period in the treatment of pT3a/b RCC and has no adverse effect on mid‐term oncologic outcomes.^169^ In recent years, despite an increase in the use of robot‐assisted radical nephrectomy (RARN) in RCC, the advantages of RARN over LRN for RCC remain controversial. Two studies showed no substantial differences in perioperative outcomes, including operative time, bleeding, conversion rate, complications, and local recurrence rates.^170^, ^171^ For PN, laparoscopic and robotic‐assisted surgery has significant advantages over open surgery in terms of bleeding control and shorter hospital stays.^172^ A meta‐analysis shows that robotic‐assisted PN has advantages over laparoscopic PN, including a lower conversion rate to open (*p* = 0.02) and radical surgery (*p* = 0.0006), shorter thermal ischemia time (*p* = 0.005), less change in postoperative estimated glomerular filtration rate (*p* = 0.03), and shorter hospital stays (*p* = 0.004); Although there were no significant differences between the two groups in terms of complications, postoperative serum creatinine changes (*p* = 0.65), operative time (*p* = 0.35), estimated blood loss (*p* = 0.76), and positive surgical margins (*p* = 0.75).^173^

Despite the controversy, the value of MIS is gradually being demonstrated, particularly in controlling bleeding and shortening hospital stays. This allows patients to undergo surgical treatment while minimizing harm and complications, greatly reducing resistance and anxiety associated with open surgery.

For patients with multiple RCC tumors at increased tumor risk (e.g., bilateral kidney tumors), those who have difficulty adapting to surgery due to age or poor physical condition, and those with solitary kidneys, ablation technology offers an additional option.^174^ the value of MIS is gradually being demonstrated, particularly in controlling bleeding and shortening hospital stays. This allows patients to undergo surgical treatment while minimizing harm and complications, greatly reducing resistance and anxiety associated with open surgery.^175^, ^176^ Tumor ablation techniques are minimally invasive, have shorter treatment times, are reproducible, have fewer complications, and higher success rates than traditional surgery.^177^ The effectiveness of MWA and RFA treatments is similar for tumors ≤4 cm, while CA is the best option for larger tumors (>3 cm).^178^, ^179^

### 3.2 Systemic treatment options

**FIGURE 4:**
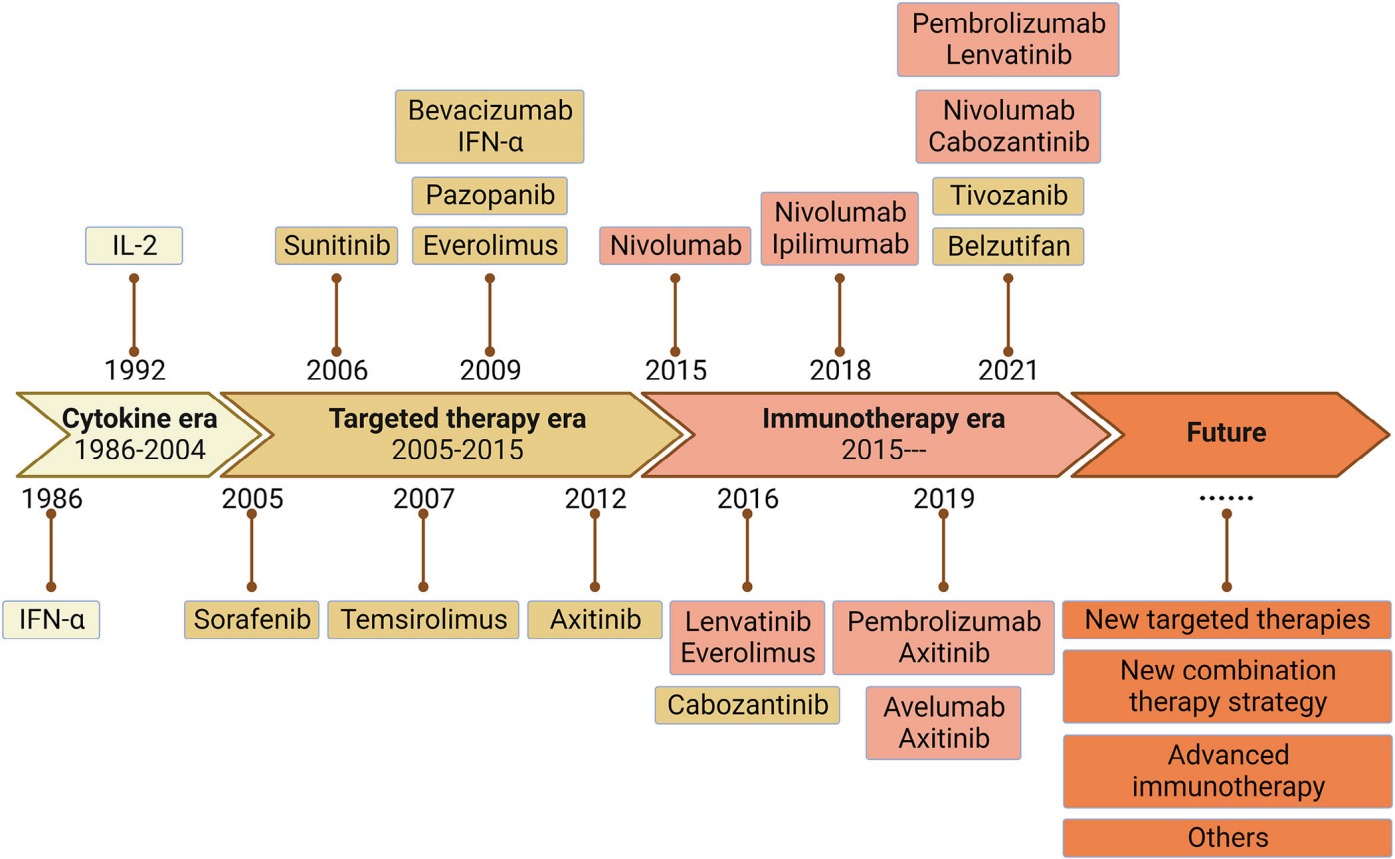
Timeline illustrating the evolving treatment landscapes and research history of renal cancer. This timeline describes the development of systemic therapies for renal cancer, exemplifying representative agents from the cytokine therapy era, the targeted therapy era, and the immunotherapy era, as well as reflections on possible future directions. This figure was created based on the tools provided by Biorender.com.

Systemic therapy for RCC mainly consists of cytokines, targeted therapies, and ICIs. Single‐class drug therapy is prone to drug resistance; thus, current research focuses on combining standard clinical therapies to resist drug resistance and expand treatment options. However, even combination therapy with existing classical therapies cannot solve all problems. Effective interventions need to be developed for patients who are insensitive to existing therapies, do not have a durable response, or fail treatment.^5^ Therefore, the continuous development of new drug targets and research into new prospective therapeutic techniques remain crucial in the field of RCC treatment, which will provide more diverse therapeutic options and contribute to the solution of the drug resistance problem and the early realization of precision medicine and individualized therapy (Figure 4).

### 3.3 Cytokine therapy

IL‐2 is a key cytokine used in RCC treatment. In 1992, high‐dose IL‐2 became the first cytokine approved for treating metastatic RCC.^180^ IL‐2 promotes the expansion of effector T‐cells, enhances their function, and improves T‐cell viability. However, it can also exert immunosuppressive effects. The mode of action differs by dose, and the effect of IL‐2 treatment varies significantly with different doses. Studies have shown that low‐dose IL‐2 specifically activate T‐reg cells and enhance their function,^181^ exerting an immunosuppressive effect to regulate chronic inflammation and autoimmune diseases.^182^ And high doses of IL‐2 can significantly stimulate NK cell and effector T cell responses, activating immune activity and playing an antitumor role.^183^ Although high‐dose IL‐2 significantly improves RCC efficacy, it can damage organs, causing multiple adverse events and even patient death.^184^ This severely limits the clinical use of *IL‐2*. Currently, several new attempts to modify *IL‐2* have yielded promising results. For example, a pegylated form of *IL‐2*, NKTR‐214 binds more favorably to *IL‐2R* β/γ, improving CD8+ T cell activation and immunoreactivity. More importantly, polyethylene glycolization improves IL‐2′s pharmacokinetics, eliminating the toxic reactions associated with high doses.^185^ A study has shown that Bempegaldesleukin (NKTR‐214) plus nivolumab demonstrates preliminary antitumor activity as first‐line therapy in advanced ccRCC patients and is well tolerated.^186^

Unlike IL‐2, interferon‐α (*IFN*‐α) can demonstrate antitumor efficacy at low doses and inhibit tumor progression by modulating tumor immunity, inhibiting tumor angiogenesis, proliferation, and differentiation.^187^ However, studies show that *IFN‐α* alone does not achieve satisfactory results in RCC, but its efficacy can be significantly improved when combined with antiangiogenic drugs or *mTOR* inhibitors.^188^

Other cytokines such as tumor necrosis factor‐α (*TNF*‐α) also play a role in treating advanced RCC, but their antitumor effects are unsatisfactory, and their systemic toxicity should not be ignored.^189^ Although TNF‐α mutants with lower systemic toxicity and higher efficiency can be obtained through genetic modification,^190^ the future prospects require further observation.

Overall, cytokine therapy for advanced, metastatic RCC has achieved limited efficacy. Although high‐dose *IL‐2* and *IFN‐α* were once standard care for metastatic RCC, the 5‐year survival rate remains low. As understanding of RCC tumor biology and pathogenesis increases, cytokine therapy is gradually being replaced by more efficient therapies.

### 3.4 Target therapy in RCC

RCC is closely associated with *VHL* mutations, which leads to an increase in the activity of its downstream the *HIF* and ultimately leads to elevated *VEGF* expression, promoting tumor angiogenesis.^191^, ^192^ Besides, the *mTOR* pathway also plays an important role in activating *HIF*.^193^ Therefore, targeted therapeutic strategies for RCC are mainly focused on targeting the *VEGF* and *mTOR* pathways.

#### 3.4.1 Tyrosine kinase inhibitors

TKIs are classical RCC therapeutic agents that primarily target RTKs, inhibiting tumor angiogenesis and growth.^194^, ^195^ Approved TKIs for RCC include sunitinib, pazopanib, axitinib, lenvatinib, and cabozantinib. Sorafenib and tivozanib are primarily used for second‐line treatment of ccRCC, and erlotinib is used primarily for pRCC (Table 1).^163^, ^196^, ^197^

**TABLE 1:** Ongoing clinical trials on targeted therapies.

categories	Agents	Target	Object	Phase	Status	Register ID
TKI	Sunitinib	VEGFR2, PDGFRβ	aRCC/mRCC	II	Active	NCT02689167
	Pazopanib	VEGFR1‐3, PDGFR, FGFR, c‐Kit, c‐Fms, CSF1R	mRCC	III	Active	NCT01575548
	Pazopanib	VEGFR1‐3, PDGFR, FGFR, c‐Kit, c‐Fms, CSF1R	mnccRCC	III	Active	NCT01767636
	Cabozantinib	MET, VEGFR1‐3, ROS1, RET, AXL, NTRK, c‐Kit	mRCC	II	Recruiting	NCT05263050
	Cabozantinib	MET, VEGFR1‐3, ROS1, RET, AXL, NTRK, c‐Kit	accRCC	II	Active	NCT04022343
	Cabozantinib	MET, VEGFR1‐3, ROS1, RET, AXL, NTRK, c‐Kit	mRCC	II	Recruiting	NCT03967522
	Cabozantinib	MET, VEGFR1‐3, ROS1, RET, AXL, NTRK, c‐Kit	aRCC/mRCC	II	Active	NCT03945773
mTOR inhibitor	Everolimus	mTOR	Renal Cancer	III	Active	NCT01120249
HIF inhibitor	Belzutifan	HIF‐2α	aRCC	III	Active	NCT04195750
	PT2385	HIF‐2α	accRCC	I	Active	NCT02293980
	PT2385	HIF‐2α	ccRCC	II	Active	NCT03108066
	Belzutifan	HIF‐2α	accRCC	I	Active	NCT04846920
	Belzutifan	HIF‐2α	accRCC	II	Active	NCT04489771
	Belzutifan	HIF‐2α	VHL‐RCC	II	Active	NCT03401788
AKT inhibitor	MK‐2206	AKT	RCC	I	Active	NCT01480154
c‐Met inhibitor	Savolitinib	c‐Met	PRCC	III	Recruiting	NCT05043090
p53MVA vaccine	p53MVA	P53	RCC	I	Active	NCT02432963
SBRT	SABR	/	oligometastatic RCC	II	Active	NCT02956798
	SABR	/	RCC	II	Recruiting	NCT03747133

aRCC/mRCC, advanced or metastatic renal cell carcinoma; ccRCC, clear cell renal cell carcinoma; nccRCC, nonclear cell renal cell carcinoma; VHL‐RCC, Von Hippel Lindau disease‐associated renal cell carcinoma; PRCC, papillary renal cell carcinoma.

*Data sources*: clinical registration website (https://clinicaltrials.gov).

Compared with IFN‐α, sunitinib monotherapy significantly prolonged progression‐free survival (PFS) and OS.^198^ Pazopanib significantly improved PFS in both treatment‐naive and cytokine‐treated mRCC and had better quality of life (QoL) compared with sunitinib, making it a potential alternative.^199^ Axitinib inhibits VEGFR1, 2, and 3, making it more effective than sorafenib, especially for patients who progressed after sunitinib treatment, with PFS of 4.8 months compared with sorafenib's 3.4 months.^200^ Today, significant progress has been made in the development of multitarget TKI drugs. For example, lenvatinib is a multiple receptor TKI that demonstrates potent antiangiogenic properties via targeting the *VEGFR*, fibroblast growth factor receptors (*FGFR*1–4), re‐arranged during transfection, platelet growth factor receptor (*PDGFRα*), and stem cell factor receptor preventing tumor angiogenesis and the further proliferation of malignant cells.^201^, ^202^, ^203^ Studies on its efficacy in RCC have focused on its combination with other drugs. A randomized phase II trial showed that lenvatinib plus everolimus significantly prolonged PFS compared with everolimus and lenvatinib alone (median 14.6 vs. 5.5 and 7.4 months).^204^ Cabozantinib, another novel TKI targeting *MET*, *VEGF*, and *AXL*, plays an active therapeutic role in RCC patients resistant to VEGFR and mTOR inhibitors.^205^, ^206^ In advanced RCC patients who progressed after previous VEGFR‐TKI treatment, cabozantinib improved median OS (21.4 vs. 16.5 months) and PFS (7.4 vs. 3.9 months), and objective response (17 vs. 3%).^207^ Compared with sunitinib, cabozantinib significantly improved median PFS (8.2 vs. 5.6 months) and objective response (46 vs. 18%) in patients with RCC of intermediate or poor risk.^208^ Beyond these, as a monoclonal VEGF antibody, it has been demonstrated that bevacizumab plus IFN is active as first‐line treatment in intermediate or poor‐risk RCC patients.^188^ Bevacizumab combined with atezolizumab prolonged PFS compared with sunitinib (11.2 vs. 7.7 months) in PD‐L1 positive mRCC patients and showed a favorable safety profile, indicating promising therapeutic prospects for bevacizumab.^209^

Targeted therapeutic agents represented by TKIs have driven the continuous development of renal cancer treatment, but the adverse effects of the drugs brought by them can still not be ignored. The targets of TKIs are many different, but in general, these drugs lead to similar adverse reactions, including systemic damage (such as rash, hypertension), digestive adverse effects (such as hepatotoxicity, anorexia, nausea, vomiting, diarrhea, constipation), and mucous membrane damage (such as hand‐foot syndrome, oral ulcers, anal ulcers, rectal fistula).^210^ Importantly, the incidence of serious adverse events remains high, which can lead to limited use of the drug. For example, in one study, 78% of patients treated with sunitinib experienced grade 3−5 serious adverse events.^211^ In addition, drug tolerance is another important issue encountered with the use of TKIs. Various possible mechanisms have been proposed to explain this phenomenon, such as (a) decreased drug bioavailability due to lysosomal chelation of the drug; (b) increased EMT due to tumor auto invasiveness; (c) increased envelope coverage of tumor vessels; (d) increased recruitment of myeloid‐derived proangiogenic inflammatory cells; and (e) drug tolerance due to single‐nucleotide polymorphisms and miRNAs.^194^ However, it is undeniable that there is still no perfect strategy to address drug resistance to targeted therapy in renal cancer. Therefore, monotherapy with TKIs is currently limited to specific patients, such as the use of cabozantinib monotherapy for those who wish to avoid the potential toxicity of immunotherapy, or have relative contraindications to immunotherapy, as well as those who prefer oral therapy.^210^

#### 3.4.2 mTOR inhibitors

Normally, *VHL* inhibits *mTORC1* signaling by regulating the degradation of mTOR pathway‐related proteins, whereas in RCC, *VHL* defects enhanced *mTOR* pathway signaling.^26^ The increased expression of *HIF* activates the *AKT*–*mTOR* pathway and enhances the antiapoptotic ability of tumor cells. The activation of the mTOR pathway further increases *HIF* expression, promoting tumor progression.^210^, ^211^ Based on this, targeting the *AKT*/*mTOR* pathway is an alternative approach to inhibit RCC progression, and numerous *mTOR* inhibitors have been shown efficacy (Table 1).

Three types of *mTOR* inhibitors have been identified: first‐generation noncompetitive inhibitors (allosteric inhibitors) that inhibit only *mTORC1*; second‐generation ATP‐competitive inhibitors that inhibit both *mTORC1* and *mTORC2*; and third‐generation bi‐steric inhibitors that also suppress only *mTORC1*.^212^

Everolimus and temsirolimus are first‐generation *mTOR* inhibitors and are the most common in RCC.^213^ Everolimus has been approved for patients who failed or are intolerant of anti‐*VEGFR* treatment, with a median PFS of 4 months compared with 1.9 months for placebo.^213^ Temsirolimus has better effects on advanced RCC compared with IFN‐α, with increased OS (10.9 vs. 8.9 months) and PFS (3.8 vs. 1.9 months).^214^ Temsirolimus is effective in treating metastatic nccRCC with poor prognosis. A study enrolled 44 patients with metastatic/recurrent nccRCC, including pRCC, chRCC, cdRCC, p11.2 translocation, and other subtypes. The median OS and PFS were 17.6 and 7.6 months, respectively. The ORR was 11% and the disease control rate was 83%, suggesting that temsirolimus was well tolerated with manageable adverse events, and was beneficial for patients with low‐risk nccRCC and effective in Asian intermediate‐ or moderate‐risk populations.^215^

Nonetheless, mTORC2 is thought to be involved in drug tolerance and cannot be fully inhibited by mTORC1 inhibitors, making second‐generation ATP‐competitive inhibitors useful as they inhibit both *mTORC1* and *mTORC2*, although these drugs remain unapproved.^216^ AZD8055, sapanisertib and vistusertib are being investigated for RCC treatment and show significant tumor‐suppressive effects..^217^, ^218^, ^219^ However, the toxic effects of ATP‐competitive inhibitors cannot be ignored. Therefore, third‐generation mTOR inhibitors selectively inhibit *mTORC1* and enhance its inhibition through spatial configuration.^220^ These inhibitors produce a more sustained effect on the target and are thus more valuable for future clinical therapy.^221^

### 3.5 Immune therapy in RCC

Previous immunotherapies have focused on cytokine therapy, including *IL‐2*,^180^ *IFN‐α*,^187^ and *TNF‐α*.^189^ Studies on immune checkpoints have revolutionized the understanding of tumor immune escape mechanisms and improved immunotherapy. With the discovery of immune checkpoints like *CTLA4*, *PD‐1*, *PD‐L1*, and new targets like *LAG‐3* in tumor progression, many ICIs have shown significant antitumor effects (Table 2).^222^

**TABLE 2:** Ongoing clinical trials on immunotherapy and combination therapy in RCC.

Agents	Combination	Target	Object	Phase	Status	Register ID
Nivolumab	/	PD1	accRCC/mccRCC	III	Active	NCT04810078
Nivolumab	/	PD1	mRCC	II	Active	NCT03126331
Nivolumab	/	PD1	RCC	III	Active	NCT03055013
Pembrolizumab	/	PD1	RCC	III	Active	NCT03142334
Pembrolizumab	/	PD1	mRCC	II	Recruiting	NCT05578664
Relatlimab	/	LAG‐3	ccRCC	II	Recruiting	NCT05148546
Nivolumab	Axitinib	PD1/VEGFR1‐3, PDGFRβ, c‐Kit	aRCC	I/II	Recruiting	NCT03172754
Nivolumab	Ipilimumab	PD1/CTLA‐4	aRCC/mRCC	III	Active	NCT02231749
Nivolumab	Cabozantinib	PD1/MET, VEGFR1‐3, ROS1, RET, AXL, NTRK, c‐Kit	aRCC/mRCC	III	Active	NCT03141177
Nivolumab	Cabozantinib	PD1/MET, VEGFR1‐3, ROS1, RET, AXL, NTRK, c‐Kit	anccRCC/mnccRCC	II	Active	NCT03635892
Nivolumab	Ipilimumab/Cabozantinib	PD1/CTLA‐4/MET, VEGFR1‐3, ROS1, RET, AXL, NTRK, c‐Kit	mRCC	II	Recruiting	NCT05048212
Nivolumab	Ipilimumab	PD1/CTLA‐4	mRCC	II	Active	NCT03297593
Nivolumab	Ipilimumab	PD1/CTLA‐4	aRCC	IIIb	Active	NCT03873402
Nivolumab	Ipilimumab	PD1/CTLA‐4	anccRCC	II	Active	NCT03075423
Nivolumab	Ipilimumab	PD1/CTLA‐4	aRCC	IV	Active	NCT04513522
Nivolumab	Bevacizumab/ipilimumab	PD1/VEGF/CTLA‐4	mRCC	I	Active	NCT02210117
Nivolumab	Ipilimumab	PD1/CTLA‐4	mRCC	III	Recruiting	NCT03793166
Pembrolizumab	Axitinib	PD1/VEGFR1‐3, PDGFRβ, c‐Kit	aRCC/mRCC	II	Recruiting	NCT04370509
Pembrolizumab	Axitinib	PD1/VEGFR1‐3, PDGFRβ, c‐Kit	aRCC/mRCC	III	Active	NCT02853331
Pembrolizumab	Lenvatinib	PD1/VEGFR1‐3, FGFR, PDGFRα, c‐Kit, RET	mnccRCC	II	Active	NCT04704219
Pembrolizumab	Lenvatinib	PD1/VEGFR1‐3, FGFR, PDGFRα, c‐Kit, RET	III‐IV RCC	II	Recruiting	NCT05485896
Pembrolizumab	Cabozantinib	PD1/MET, VEGFR1‐3, ROS1, RET, AXL, NTRK, c‐Kit	mRCC	I/II	Active	NCT03149822
Atezolizumab	Bevacizumab	PDL1/VEGF	anccRCC	II	Active	NCT02724878
Atezolizumab	Cabozantinib	PDL1/MET, VEGFR1‐3, ROS1, RET, AXL, NTRK, c‐Kit	aRCC/mRCC	III	Active	NCT04338269
Avelumab	Axitinib	PDL1/VEGFR1‐3, PDGFRβ, c‐Kit	mRCC	II	Recruiting	NCT04698213
Avelumab	Axitinib	PDL1/VEGFR1‐3, PDGFRβ, c‐Kit	aRCC	III	Active	NCT02684006
Toripalimab	Axitinib	PD1/VEGFR1‐3, PDGFRβ, c‐Kit	aRCC/mRCC	III	Active	NCT04394975
Tislelizumab	Lenvatinib	PD1/VEGFR1‐3, FGFR, PDGFRα, c‐Kit, RET	III‐IV RCC	II	Recruiting	NCT05485883
Cadonilimab	Lenvatinib	PD1, CTLA‐4/VEGFR1‐3, FGFR, PDGFRα, c‐Kit, RET	accRCC/mccRCC	II	Recruiting	NCT06035224
Cadonilimab	Lenvatinib	PD1, CTLA‐4/VEGFR1‐3, FGFR, PDGFRα, c‐Kit, RET	ccRCC	II	Recruiting	NCT06138496
Axitinib	Nivolumab	VEGFR1‐3, PDGFRβ, c‐Kit/PD1	mRCC	II	Recruiting	NCT05817903
Axitinib	Pembrolizumab	VEGFR1‐3, PDGFRβ, c‐Kit/PD1	mPRCC	II	Recruiting	NCT05096390
Axitinib	Nivolumab	VEGFR1‐3, PDGFRβ, c‐Kit/PD1	tRCC	II	Active	NCT03595124
Lenvatinib	Everolimus/Pembrolizumab	VEGFR1‐3, FGFR, PDGFRα, c‐Kit, RET/mTOR/PD1	aRCC/mRCC	III	Active	NCT02811861
Lenvatinib	Everolimus	VEGFR1‐3, FGFR, PDGFRα, c‐Kit, RET/mTOR	RCC	II	Active	NCT03173560
Lenvatinib	Pembrolizumab	VEGFR1‐3, FGFR, PDGFRα, c‐Kit, RET/PD1	mnccRCC	II	Recruiting	NCT04267120
Lenvatinib	Pembrolizumab	VEGFR1‐3, FGFR, PDGFRα, c‐Kit, RET/PD1	anccRCC	II	Recruiting	NCT04393350
Lenvatinib	Everolimus	VEGFR1‐3, FGFR, PDGFRα, c‐Kit, RET/mTOR	mRCC	II	Recruiting	NCT05012371
Pazopanib	Bevacizumab	VEGFR1‐3, PDGFR, FGFR, c‐Kit, c‐Fms, CSF1R/VEGF	mccRCC	I/II	Recruiting	NCT01684397
Cabozantinib	Nivolumab/ipilimumab	MET, VEGFR1‐3, ROS1, RET, AXL, NTRK, c‐Kit/PD1/CTLA‐4	aRCC/mRCC	III	Active	NCT03937219
Cabozantinib	Atezolizumab	MET, VEGFR1‐3, ROS1, RET, AXL, NTRK, c‐Kit/PDL1/CTLA‐4	apRCC	II	Recruiting	NCT05411081
Cabozantinib	Nivolumab/ipilimumab	MET, VEGFR1‐3, ROS1, RET, AXL, NTRK, c‐Kit/PD1/CTLA‐4	anccRCC	II	Recruiting	NCT04413123
Tivozanib	Nivolumab	VEGFR1‐3/PD1	RCC	III	Active	NCT04987203
Sitravatinib	Nivolumab/ipilimumab	AXL, MER, VEGFR2, PDGFR, c‐Kit, RET, MET, DDR2, TRKA/PD1/CTLA‐4	accRCC/mccRCC	I/Ib	Active	NCT04518046
Sitravatinib	Nivolumab	AXL, MER, VEGFR2, PDGFR, c‐Kit, RET, MET, DDR2, TRKA/PD1	mccRCC	II	Active	NCT04904302
XL092	Nivolumab	MET, VEGFR2, AXL, MER/PD1	anccRCC/mnccRCC	III	Recruiting	NCT05678673
Belzutifan	Lenvatinib/pembrolizumab	HIF‐2α/VEGFR1‐3, FGFR, PDGFRα, c‐Kit, RET/PD1	aRCC	I	Active	NCT05030506
Belzutifan	Lenvatinib	HIF‐2α/VEGFR1‐3, FGFR, PDGFRα, c‐Kit, RET	aRCC	III	Active	NCT04586231
Belzutifan	Cabozantinib	HIF‐2α/MET, VEGFR1‐3, ROS1, RET, AXL, NTRK, c‐Kit	accRCC	II	Active	NCT03634540
Belzutifan	Pembrolizumab	HIF‐2α/PD1	ccRCC	III	Recruiting	NCT05239728
Belzutifan	Palbociclib	HIF‐2α/CDK4/6	accRCC	I/II	Recruiting	NCT05468697
NKT2152	Palbociclib/sasanlimab	HIF‐2α/CDK4/6/PD1	accRCC/mccRCC	II	Recruiting	NCT05935748
Abemaciclib	Sunitinib	CDK4/6/VEGFR2, PDGFRβ	mRCC	Ib	Active	NCT03905889
Bempegaldesleukin	Nivolumab/TKI	IL‐2/PD1/VEGFR	aRCC/mRCC	I	Active	NCT04540705
IL‐2	SBRT	/	mRCC	II	Active	NCT02306954
SBRT	Ipilimumab/Nivolumab	CTLA‐4/PD1	mRCC	II	Recruiting	NCT04090710
CBM588	Nivolumab/Cabozantinib	Gut microbiota/PD1/MET, VEGFR1‐3, ROS1, RET, AXL, NTRK, c‐Kit	mRCC	I	Active	NCT05122546
CBM588	Nivolumab/ipilimumab	Gut microbiota/PD1/CTLA‐4	mRCC	I	Active	NCT03829111
Dendritic Cell Vaccine	Autologous α‐DC1/TBVA vaccine	/	ccRCC	IIA	Recruiting	NCT05127824
CAR‐T Therapy	CD70 CAR‐T cells	/	aRCC	I	Recruiting	NCT05420519
	CAIX CAR‐T cells	/	aRCC	I/II	Recruiting	NCT04969354
	ALLO‐316	/	accRCC/mccRCC	I	Recruiting	NCT04696731
	CTX130	/	ccRCC	I	Active	NCT04438083

aRCC/mRCC, advanced or metastatic renal cell carcinoma; ccRCC, clear cell renal cell carcinoma; nccRCC, nonclear cell renal cell carcinoma; VHL‐RCC, Von Hippel–Lindau disease‐associated renal cell carcinoma; tRCC, TFE/translocation renal cell carcinoma.

*Data sources*: clinical registration website (https://clinicaltrials.gov).

#### 3.5.1 Anti‐CTLA4

Cytotoxic T‐lymphocyte‐associated antigen 4 (*CTLA‐4*), the first immune checkpoint receptor on activated effector T cells, blocks downstream intracellular signaling upon binding to corresponding ligands. This inhibits T cell proliferation and decreases T cell activation, ultimately leading to immunosuppression.^223^ Therefore,*CTLA‐4* inhibitors like ipilimumab and tremelimumab can inhibit tumor immune escape by blocking *CTLA‐4* and enhancing T cell infiltration and activation in RCC. However, immune‐related adverse events like enteritis caused by anti‐*CTLA‐4* antibodies have severely limited their clinical application in RCC treatment.^224^ Therefore, much of the research on *CTLA‐4* antagonists has focused on their combination with other agents like anti‐*PD‐1*, as described in later sections.

#### 3.5.2 Anti‐PD‐1/PD‐L1

Another beneficial immune checkpoint is *PD‐1*/*PD‐L1*. The anti‐*PD‐1* antibody nivolumab was approved in 2015 for treating metastatic RCC patients who failed TKI therapy. Studies have shown that in metastatic RCC patients who received 1 or 2 antiangiogenic regimens, anti‐*PD‐1* antibody nivolumab significantly prolonged median OS compared with the mTOR inhibitor everolimus (25.8 vs. 19.7 months), demonstrating superior efficacy in treating *VEGF*‐refractory RCC.^225^ For both advanced ccRCC and nccRCC, single‐agent pembrolizumab showed promising antitumor activity as a first‐line treatment.^226^ In a phase II prospective study of advanced nccRCC, first‐line pembrolizumab monotherapy showed promising antitumor activity with a median OS was 28.9 months, a 24‐month rate was 58.4% and an ORR was 26.7% in all patients.^227^ Similar to anti‐PD‐1 therapy, anti‐PD‐L1 antibodies like atezolizumab have demonstrated promising antitumor activity manageable safety profile in the treatment of progressive RCC.^228^ The combination of atezolizumab and bevacizumab demonstrated a high objective response (49 vs. 14%) and significantly improved patient symptoms and prolonged median PFS (8.3 vs. 5.3 months), especially for PD‐L1‐positive tumors, compared with sunitinib.^227^

#### 3.5.3 Anti‐LAG3

Lymphocyte activation gene 3 (*LAG‐3*), also known as *CD223*, is expressed on activated human T cells, NK cells, B cells, and DCs.^229^, ^230^ As an inhibitory receptor, *LAG‐3* exerts negative regulatory effects on T cells and mediates T cell depletion in association with *PD‐1*.^231^ *LAG‐3* binds to human leukocyte antigen class II molecules and activates DCs, increasing their antigen‐presenting capacity and facilitating antigen presentation to CD8+ T cells.^232^ In RCC, blocking *PD‐1* leads to upregulation of *LAG‐3* expression, while simultaneous inhibition of *PD‐1* and *LAG‐3* increases *IFN‐γ* release, suggesting that combined inhibition of *PD‐1* and *LAG‐3* is a promising checkpoint blockade combination.^233^

### 3.6 Combined therapy

Recent studies have revealed a link between *VHL–HIF‐1–VEGF* signaling‐mediated tumor angiogenesis and changes in the TME. For instance, *HIF‐1α*‐induced *VEGF‐a* promotes CD8+ T cell infiltration by regulating endothelial permeability and maintaining the effector state of CD8+ T cells.^234^ Inhibition of *HIF1‐α* reduced *PD‐L1* expression in a mouse tumor model.^235^ These studies suggest the potential of combining TKIs and ICIs for RCC treatment. Indeed, combining these agents significantly improved survival compared with sunitinib alone.^236^ Combination therapy is now the recommended first‐line treatment for advanced, metastatic RCC (Table 2).^163^

Given the multitude of targeted agents and ICIs for RCC treatment, clinical studies of combination therapies have attracted much attention. For TKI and immunotherapy combination, a study showed that in previously untreated advanced RCC patients, pembrolizumab plus axitinib resulted in significantly longer PFS (15.1 vs. 11.1 months) and higher ORR (59.3 vs. 35.7%) than sunitinib.^236^ Similarly, another phase III trial has revealed that nivolumab plus cabozantinib was more effective than sunitinib in previously untreated advanced RCC patients, with median OS of 37.7 months in the combination group and 34.3 months in the sunitinib group. Updated median PFS was 16.6 versus 8.3 months.^237^

Additionally, in advanced RCC patients, durable clinical benefits were observed with nivolumab plus ipilimumab versus sunitinib at 5 years, with median OS of 55.7 versus 38.4 months and objective response of 39.3 versus 32.4%.^238^ An ongoing phase III b trial (NCT03873402) is testing the efficacy and safety of nivolumab plus ipilimumab versus nivolumab monotherapy in previously untreated advanced and intermediate‐ or low‐risk RCC. Recent studies have also explored the efficacy and safety of three‐drug combination therapies. In a phase III, double‐blind trial, 855 previously untreated advanced ccRCC patients with intermediate or poor prognostic risk were randomly assigned to receive either 40 mg of cabozantinib per day with nivolumab and ipilimumab (experimental group) or a matching placebo with nivolumab and ipilimumab (control group). An interim analysis supported a better ORR for patients receiving triple therapy (43 vs. 36%). However, patients receiving triple therapy had higher grade ≥3 toxicity (73 vs. 41%) and more treatment interruptions (12 vs. 5%) compared with doublet therapy controls. This result supports that cabozantinib combined with nivolumab and ipilimumab is more effective and significantly prolongs PFS compared with nivolumab and ipilimumab alone in previously untreated advanced RCC patients with intermediate or poor prognostic risk. However, safety concerns remain, as grade 3 or 4 adverse events were more common in the experimental group.^239^

These results suggest that combination therapy is significantly superior to monotherapy, both for targeted therapy with immunotherapy and for combining different ICIs.

### 3.7 CAR‐T therapy

RCC is infiltrated with many T cells, but these T cells do not always exert antitumor effects due to numerous inhibitory factors.^240^ Therefore, restoring T cell function in RCC is a valuable therapeutic strategy, including CAR‐T cell therapy, which uses bioengineering to highly express CAR on T cells, enabling them to exert antitumor effects by interacting with tumor target antigens.^241^ The striking response of CAR‐T cell therapy in hematologic malignancies has drawn attention to its use in solid tumors, including RCC.^242^ It was first validated in a phase I/II trial against carboxy‐anhydrase‐IX (*CAIX*), a gene regulated by *HIF* and upregulated in RCC, but no significant therapeutic response was found.^243^ Encouragingly, the combination with sunitinib demonstrated a favorable antitumor response in animal RCC models, suggesting that sunitinib's immunomodulatory effects may enhance the efficacy of CAR‐T cell therapy.^244^

Additionally, clinical studies of CAR‐T cell therapy targeting *CD70*, *AXL*, and *ROR2* are in progress, and these may represent the next generation of RCC therapies. A phase I dose‐escalation study (NCT04696731) is evaluating the safety, efficacy, and cell kinetics of ALLO‐316 (targeting *CD70*) in adults with advanced or metastatic ccRCC following a lymphodepleting regimen that includes fludarabine, cyclophosphamide, and ALLO‐647 (targeting *CD52*) to determine the phase II dose. Another phase I dose escalation and cohort expansion study (NCT04438083) is now assessing the safety and efficacy of CTX130, an allogeneic CRISPR–Cas9‐engineered T cell targeting *CD70*, in subjects with advanced, recurrent, or refractory RCC. *ROR2* and RTK *AXL* are highly expressed in RCC, correlate with poor prognosis, and are valuable therapeutic targets.^86^, ^245^ A phase I/II trial (NCT03393936) is evaluating the safety, tolerability, and antitumor activity of T‐cell infusion for treating adults with relapsed and refractory stage IV metastatic RCC. *ROR2*‐positive RCC subjects will receive CCT301‐59 T‐cells, while *AXL*‐expressing RCC subjects will receive CCT301‐38 T‐cell therapy. These studies will help us understand the value of CAR‐T cell therapy, potentially improving RCC treatment options (Table 2).

### 3.8 DCs vaccine

As major antigen‐presenting cells, DCs activate T cells by expressing various costimulatory molecules..^246^ Thus, autologous DC vaccines can stimulate tumor‐specific immune responses by exposing tumor antigens to DCs.^247^ Several clinical studies are currently validating the responsiveness and safety of DC vaccines in the treatment of RCC. For example, a phase II trial (NCT04203901) is investigating the efficacy of an autologous tumor antigen‐loaded DC vaccine, CMN‐001, combined with ipilimumab/nivolumab (I/N) in patients with intermediate/low‐risk mRCC compared with I/N alone. The safety of a DC‐tumor fusion vaccine with granulocyte macrophage colony stimulating factor is being tested in an active phase I/II trial (NCT00458536) in 38 patients with mRCC who underwent reduced‐volume nephrectomy and had not received systemic therapy. And a phase II trial (NCT05127824) is evaluating the role of neoadjuvant autologous tumor vascular antigen DCs vaccine injections intradermally with cabozantinib prior to surgical resection in patients with localized RCC. These studies will soon provide definitive evidence about DC vaccine treatment for RCC (Table 2).

### 3.9 CDKs inhibitor

CDKs are serine/threonine kinases that promote DNA synthesis and chromosome segregation by regulating substrate phosphorylation, thereby controlling the cell cycle and participating in key processes like gene transcription, insulin secretion, glycogen synthesis, and neuronal function.^248^, ^249^ Consequently, CDK inhibitors have been developed to treat various diseases caused by CDK abnormalities. CDK has long been an attractive target for cancer therapy due to defective cell cycle and proliferation regulation in most tumors, leading to the development and testing of many CDK inhibitors.^250^ However, only a few CDK inhibitors have shown potential therapeutic value for RCC. Ribociclib, a *CDK4/6* inhibitor, has been shown to inhibit RCC proliferation and induce apoptosis in vivo and in vitro, acting synergistically with chemotherapeutic or immunotherapeutic agents without damaging normal renal cells and fibroblasts.^251^ Synergistic effects of *CDK4/6* inhibitors with *HIF‐2α* inhibitors in *VHL*‐deficient RCC have also been identified.^252^ The safety, tolerability, and maximum tolerated dose of the oral combination of Abemaciclib and sunitinib in patients with advanced and metastatic RCC are being evaluated in a study (NCT03905889). A phase II study (NCT05935748) is evaluating the safety, efficacy, and pharmacokinetics of the *HIF‐2α* inhibitor NKT2152 in combination with palbociclib (doublet) and the *PD‐1* inhibitor sasanlimab (triplet) in subjects with advanced or metastatic ccRCC who have received prior therapy to determine the recommended dose expansion (Table 1).

### 3.10 HIF inhibitor

*HIF‐α* enhances tumor cell growth and resistance to treatment by regulating the expression of downstream target genes such as *VEGF* and glucose transporter protein‐1, improving the angiogenic and glycolytic capacity of tissues.^253^ Of the three HIFs, *HIF‐2α* is considered the major RCC promoter, making it a potential therapeutic target.^254^ The first‐generation *HIF‐2α* inhibitor PT2385 demonstrated a favorable safety profile in a phase I dose‐escalation trial involving patients with metastatic ccRCC who had undergone extensive pretreatment. Moreover, 52% of patients were stable, 12% had a partial response, and 2% had a complete response.^255^ Although this novel drug has promising efficacy in heavily pretreated mRCC patients, variability in drug exposure is a concern, leading to underdosing in some patients and inadequate drug exposure. The second‐generation *HIF‐2α* inhibitor belzutifan (MK‐6482) is more selective. In a single‐arm phase II study, 61 patients with VHL disease‐associated localized RCC and pancreatic lesions were treated with 120 mg of belzutifan daily, showing activity in RCC and non‐RCC neoplasms associated with VHL disease.^256^ An ongoing open‐label, single‐arm, phase II study (NCT03634540) demonstrates promising antitumor activity of belzutifan plus cabozantinib in patients with pretreated ccRCC.^257^

*HIF‐2α* promotes the formation of an immunosuppressive TME through multiple pathways, such as promoting the development and proliferation of Tregs,^258^ upregulating the expression of stem cell factors, thereby inducing the secretion of *TGF*‐β and *IL‐10*,^259^ and *HIF‐2α* expression correlates with an increase in the expression of PD‐L1 on ccRCC tumor cells, which indirectly inhibits T cell function.^260^ These results provide a strong basis for the use of *HIF‐2α* inhibitors in combination with checkpoint inhibitors. ccRCC preclinical data suggest that the combination of *HIF‐2α* inhibitors and checkpoint inhibitors can inhibit tumor growth by increasing T‐cell infiltration, modulating myeloid cells, and altering chemokine expression.^261^ The phase III LITESPARK‐022 study is assessing the role of belzutifan plus pembrolizumab in adjuvant therapy after nephrectomy (NCT05239728). More extensive combination therapy studies are underway. An open‐label, randomized phase III study (NCT04736706) is evaluating the efficacy and safety of pembrolizumab in combination with belzutifan and lenvatinib, or quavonlimab (*CTLA‐4* antagonist) in combination with lenvatinib, compared with pembrolizumab and lenvatinib, as first‐line treatment in participants with advanced ccRCC. The United States Food and Drug Administration has approved belzutifan for patients with VHL‐related cancers, including RCC (Table 1).^262^

### 3.11 Antibody–drug conjugates

ADCs are a novel therapeutic approach combining monoclonal antibodies (mAbs) and cytotoxic drugs via a linker. The mAb anchors the ADC to the tumor cell surface by binding to a tumor‐specific antigen (TSA), allowing the cytotoxic payload to enter the tumor cell and exert its effect.^263^ This approach leverages the specificity of mAbs and the potency of highly cytotoxic drugs to selectively target tumor cells and deliver the cytotoxic payload to the tumor site, potentially reducing drug‐induced side effects.^264^ Consequently, ADC therapies have been approved for treating various hematologic and solid tumors, including breast cancer,^265^ gastric or gastroesophageal junction adenocarcinoma,^266^ cervical cancer,^267^ ovarian cancer,^268^ and metastatic urothelial carcinoma.^269^

Although no ADC has been approved for use in RCC, numerous clinical trials are exploring various ADCs for potential applications. CDX‐014, an ADC targeting immunoglobulin mucin‐1 (*TIM‐1*), resulted in clinical benefit in 31% of patients in a study of previously treated ccRCC and pRCC, with PFS and OS of 2.7 and 12.6 months, respectively. This finding suggests that CDX‐014 has a manageable toxicity profile and early signs of activity, supporting further evaluation in patients with advanced RCC and other cancers expressing *TIM‐1*.^270^ *CD70* is another potential target that is highly expressed in hematologic and solid tumors, and correlates with poor prognosis.^271^ It binds to its receptor *CD27* and coregulates Treg mobilization or survival, leading to immunosurveillance in the TME and promoting tumor growth.^272^ Therefore, the safety and tolerability of e *CD70*‐targeted ADCs such as AMG 172,^273^ MDX‐1203,^274^ SGN‐CD70A,^275^ SGN‐75^276^ were tested in RCC and showed modest efficacy, manageable adverse effects, and favorable overall tolerability. Two drugs targeting ectonucleotide pyrophosphatase/phosphodiesterase 3, AGS‐16M8F and AGS‐16C3F, were evaluated in advanced refractory RCC. Indeed, AGS‐16C3F, an improved type of the former, was tolerated and had durable antitumor activity at 1.8 mg/kg every 3 weeks.^277^ Overall, research on ADCs in RCC is still in its early stages. More studies are needed to support ADC treatment in RCC, especially in relapsed or refractory cases. Discovering more valuable and specific targets may advance this progress (Table 2).

### 3.12 Stereotactic body radiation therapy

SBRT, also known as stereotactic ablative radiation therapy (SABR), is a precise, high‐dose radiation technique that can exert cytoreductive and immunomodulatory effects to improve survival in mRCC patients undergoing systemic therapy. It has been used for radical treatment of inoperable primary RCC and for bone and brain metastases of mRCC.^278^, ^279^ Although RCC is considered insensitive to radiotherapy, the high doses of radiation delivered by SBRT effectively control tumor growth.^280^ Analysis of resected RCC treated with SBRT by Ali et al.^281^ identified enrichment of immune response pathways, T‐cell accumulation, and dynamic remodeling of circulating T‐cell pools after treatment. Studies have shown that SBRT is safe and effective for treating locally recurrent RCC, and that localized therapy can slow disease progression compared with systemic therapy alone.^282^ Currently, attention is focused on the combination of SBRT with other therapies, especially ICI, in mRCC. A phase II randomized clinical trial (NCT04090710) is evaluating SBRT as upfront cytoreductive therapy to the primary renal mass along with combination I/N versus I/N alone therapy in patients with intermediate/poor risk mRCC who are not candidates for CN For patients with metastatic ccRCC who have failed at least one prior antiangiogenic therapy, a phase II trial (NCT02781506) is also evaluating the use of SBRT to multiple metastatic sites concurrently administered with Nivolumab. The combined therapeutic value of SBRT is expected to benefit patients with refractory RCC, such as after recurrence and metastasis (Table 2). Recent studies have further demonstrated the feasibility of using SBRT in combination with TKIs or ICIs in patients with mRCC, especially those with low metastatic tumor burden.^283^

### 3.13 Gut microbiota

The gut microbiome participates in host nutrient absorption and metabolism, maintains microcirculation, regulates immune responses, and influences tumorigenesis, progression, prognosis, and therapy through pathways like the synthesis or conversion of circulating metabolites, phagocyte activation, and production of short‐chain fatty acids.^284^, ^285^ Increasing evidence suggests that gut microbiota a key host factor associated with the response to ICI therapy in various solid tumors, including RCC.^286^ A study analyzing baseline stool samples of patients prior to ICI therapy showed that greater microbial diversity was associated with clinical benefit from ICI therapy (*p* = 0.001), and multiple species were associated with clinical benefit or lack thereof.^287^ Antibiotic use resulted in lower ORR and shorter PFS with ICI treatment and adversely affected OS in patients treated with IFN or with *VEGF*‐targeted therapies and prior cytokines.^288^ This also proves that modulation of the gut microbiota may play an important role in optimizing the prognosis of patients treated with ICIs.

Numerous studies are currently validating the potential to improve RCC treatment and prognosis through gut microbiota modulation. A phase I trial (NCT03829111) compared the effects of nivolumab and ipilimumab in treatment of RCC patients with or without daily oral CBM588, a bifidogenic live bacterial product, and found that PFS was significantly longer in patients receiving nivolumab–ipilimumab with CBM588 than without (12.7 vs. 2.5 months) and the response rate was also higher in patients receiving CBM588 (58 vs. 20%).^289^ Fecal microbiota transplantation (FMT) is another treatment option under investigation.The TACITO phase I/II trial (NCT04758507) is evaluating whether donor FMT that responds well to ICIs improves ICI efficacy in treatment‐naïve patients. Another PERFORM clinical trial (NCT04163289) is investigating whether healthy‐donor FMT can reduce the incidence of irAEs in mRCC patients receiving a combination of immunotherapies. Additionally, the development of engineered microbiomes has made it possible to induce antitumor activity using specific bacterial strains.^290^ Modulation of the gut microbiome through dietary adjustments has also been shown to kill tumor cells, reverse disease‐related symptoms, and improve survival.^291^ Overall, the gut microbiota may become future biomarkers for evaluating both the efficacy and safety of ICIs (Table 2).

## 4 FUTURE PERSPECTIVE

**FIGURE 5:**
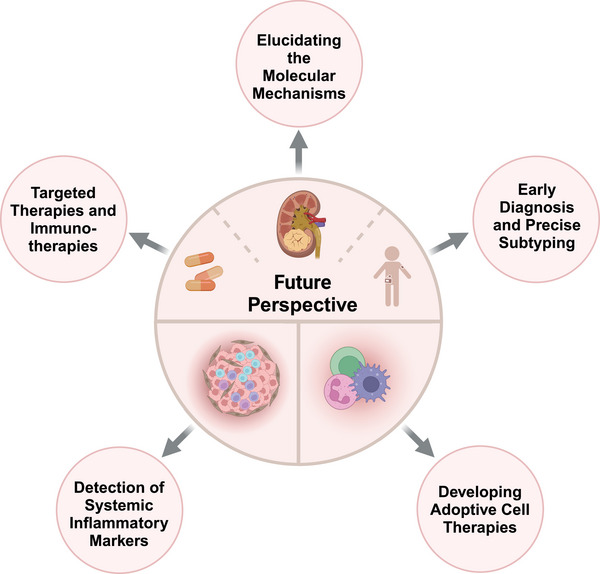
Future directions in RCC research: unraveling molecular mechanisms, devising innovative diagnostic and therapeutic approaches, exploring drug resistance and inflammation‐associated pathways, and promoting adoptive cell therapy to optimize patient outcomes. This figure was created based on the tools provided by Biorender.com.

Renal cancer is one of the most prevalent malignant neoplasms in the urinary system, with increasing incidence and mortality rates globally. Despite substantial progress in the diagnosis and treatment of renal cancer in recent years, a significant proportion of patients are still diagnosed at an advanced stage, leading to a poor prognosis. Consequently, it is of paramount importance to thoroughly elucidate the pathogenesis of renal cancer, identify novel therapeutic targets and strategies, and improve the prognosis of renal cancer patients. Future research on renal cancer should emphasize the following aspects (Figure 5).

Elucidating the molecular mechanisms of renal cancer, particularly the signaling pathways associated with invasion, progression, initiation, and proliferation, remains a critical issue that warrants further investigation.^292^, ^293^ Moreover, systematically identifying target antigens and elucidating the mechanisms of action of potential therapeutic drugs within the unique cancer‐immune cycle of renal cancer necessitates the establishment of more precise animal models and in vitro culture systems to better recapitulate the biological behavior and drug responses of human renal cancer. A comprehensive understanding of these mechanisms is pivotal for identifying novel therapeutic targets and developing precision medicine strategies.

Early diagnosis and precise subtyping of renal cancer continue to pose significant challenges. The current deficiency in highly specific and sensitive markers results in a considerable number of patients receiving a diagnosis at an advanced stage. Consequently, there is a pressing necessity to develop innovative liquid biopsy technologies and imaging techniques to facilitate noninvasive early screening and dynamic monitoring of renal cancer by detecting circulating tumor cells, cell‐free tumor DNA, exosomes, and other biomarkers. Furthermore, the integration of multiomics data and clinical information is crucial for constructing multiomics predictive models that enable precise molecular subtyping and risk assessment of renal cancer, ultimately guiding personalized treatment decisions. To address drug resistance challenges, it is imperative to identify the most appropriate patients for specific treatments and employ high‐quality biomarkers to develop personalized treatment strategies. It is expected that future prognostic biomarkers will be developed through a combination of approaches that precisely characterize the cancer microenvironment. Urinary biomarkers hold promise as a cost‐effective and user‐friendly screening tool for renal cancer in the future.

Targeted therapies and immunotherapies have made remarkable advancements in improving the prognosis of renal cancer patients; however, the development of drug resistance and the occurrence of toxic side effects continue to pose significant challenges. Going forward, it is imperative to conduct thorough investigations into drug resistance mechanisms, identify new therapeutic targets and drugs, and refine dosing regimens and combination therapy strategies. Concurrently, research on the TME should be intensified to elucidate the impact of immune cell infiltration, inflammatory factors, metabolic reprogramming, and other factors on drug efficacy, and to formulate new immunotherapy combination regimens. Furthermore, it is crucial to identify reliable biomarkers for predicting patients’ response and tolerance to treatment, thereby enabling precision medication and toxicity management.

Inflammation plays a pivotal role in the onset and progression of renal cancer; therefore, future research should prioritize investigations into inflammation‐related mechanisms and biomarkers. Detection of systemic inflammatory markers in blood and urine samples from renal cancer patients can yield valuable prognostic information. Single‐cell RNA sequencing technology facilitates the determination of the source, origin, and type of inflammation‐induced cells, the identification of drug resistance‐related pathways, and the establishment of effective risk stratification models. A comprehensive understanding of the intricate inflammatory pathways regulating tumor cell or TME plasticity might lead to the identification of novel therapeutic targets for renal cancer and lay the foundation for personalized treatment approaches.

Metabolic reprogramming, characterized by alterations in cellular metabolism to support rapid proliferation and survival, is a critical hallmark of renal cancer. Future studies should aim to elucidate the complex interplay between oncogenic signaling pathways and metabolic reprogramming in renal cancer. Elucidating the molecular mechanisms driving metabolic adaptations and identifying key metabolic vulnerabilities may pave the way for the development of novel therapeutic strategies targeting the unique metabolic dependencies of renal cancer cells.

The future of renal cancer research hinges on the integration of multiomics approaches, network analysis, and precision medicine. Comprehensive profiling of the genome, epigenome, transcriptome, proteome, and metabolome will yield unprecedented insights into the molecular mechanisms underlying renal cancer pathogenesis. Network analysis will facilitate the identification of critical signaling hubs and potential therapeutic targets. Precision medicine, guided by multiomics data and network analysis, will lay the foundation for personalized treatment strategies tailored to each patient's distinct molecular profile, ultimately enhancing patient outcomes and QoL.

Although adoptive cell therapy for renal cancer is still in its nascent stages, it is steadily gaining traction. To advance the development of this field, several critical issues must be addressed: the paucity of heterogeneously expressed TSAs with minimal off‐target toxicity, the trafficking and persistence of T cells within solid tumors, and surmounting the immunosuppressive TME. Future research should concentrate on developing innovative TSAs, optimizing T cell engineering strategies (such as chemokine receptor expression, cytokine secretion, and immune checkpoint inhibition), and investigating novel cellular therapies like CAR‐transduced macrophages. Combinatorial therapeutic approaches employing cutting‐edge cell engineering modules exhibit great potential for substantially enhancing the efficacy of adoptive cell therapy in the treatment of renal cancer.

In summary, the pathogenesis of renal cancer is complex, involving multiple signaling pathways and immunoregulatory mechanisms. Future research should aim to make significant progress in elucidating molecular mechanisms, identifying novel biomarkers, optimizing adoptive cell therapy, and investigating inflammation‐related pathways. The ultimate goal is to provide more precise and effective diagnostic and therapeutic options for renal cancer patients, thereby improving survival rates and QoL. This requires close integration of basic research and clinical practice, as well as collaborative efforts from multidisciplinary teams. Only through the continuous advancement of scientific research in the field of renal cancer can the goal of precision medicine be fully realized, enabling each renal cancer patient to receive the most appropriate personalized treatment. It is anticipated that through the concerted efforts of all stakeholders, we will undoubtedly overcome the global challenge posed by renal cancer and enable more patients to achieve long‐term survival benefits.

## AUTHOR CONTRIBUTIONS

Aimin Jiang, Jinxin Li, Ziwei He, Ying Liu, and Kun Qiao reviewed the literature and drafted the manuscript. Linhui Wang, Yu Fang, and Le Qu reviewed relevant literature. Linhui Wang, Peng Luo, and Anqi Lin designed and reviewed the manuscript. All the authors have read and approved the final version of the manuscript.

## CONFLICT OF INTEREST STATEMENT

The authors declare no conflict of interests.

## ETHICS STATEMENT

Not applicable.

## Data Availability

Not applicable.
